# Association analysis of Vascular Endothelial Growth Factor-A (VEGF-A) polymorphism in rheumatoid arthritis using computational approaches

**DOI:** 10.1038/s41598-023-47780-8

**Published:** 2023-12-11

**Authors:** Iraj Ahmed, Peter John, Attya Bhatti

**Affiliations:** 1 Atta-Ur-Rehman School of Applied Biosciences (ASAB), National University of Sciences and Technology (NUST), Islamabad, Pakistan; 2 Faculty of Applied Biosciences, ASAB, NUST, Islamabad, Pakistan

**Keywords:** Computational biology and bioinformatics, Genetics, Diseases

## Abstract

Rheumatoid arthritis (RA), is marked by joint inflammation leading to pannus formation which results in cartilage destruction promoting bone erosion. The pathological hallmark of RA includes synovial hyperplasia and synovial angiogenesis. Active tissue neovascularization is observed in RA. Vascular endothelial Growth factor A (VEGFA), an endothelial cell-specific proangiogenic molecule is triggered by hypoxic cells and its levels are upregulated in RA. The aim of this study was to investigate functional and pathogenic VEGFA variants and to identify the impact of point mutation in VEGFA’s interaction with VEGFR2 and how these polymorphisms affect the susceptibility and severity of RA. We investigated impact of these point mutations on the stability of VEGFA using various computational tools. These mutations were further identified by conservational profile as they are highly involved as structural and functional mutations. Furthermore, these selected variants were modelled and docked against targeted domain regions IGD2 and IGD3 of VEGFR2. Further molecular dynamic simulations were performed using Gromacs. Out of 168 nsSNPS, 19 were highlighted as highly pathogenic using *insilico* prediction tools. InterPro and ConSurf revealed domains and conserved variants respectively. After stability analysis, we concluded that almost all the mutations were responsible for decreasing the protein stability. HOPE predicted that all the selected damaging nsSNPs were present in the domain which is essential for the functioning of VEGFA protein. Constructed Ramachandran plot and ERRAT validated the quality of all the models. Based on the interactions predicted by STRING database, we performed Protein–Protein docking between VEGFA and VEGFR2. We found few conserved interactions and new polar contacts among wild-type and mutants with VEGFR2. From the simulations, we concluded that mutant R108Q was the most stabilizing mutant among all others whereas R82Q, C86Y, and R108W complexed with VEGFR2 were comparatively less stabilizing as compared to the wild type. This study provides insight into pathogenic nsSNPs that can affect VEGFA protein structure and function. These high-risk variants must be taken into consideration for genetic screening of patients suffering from RA.

## Introduction

**Figure 1:**
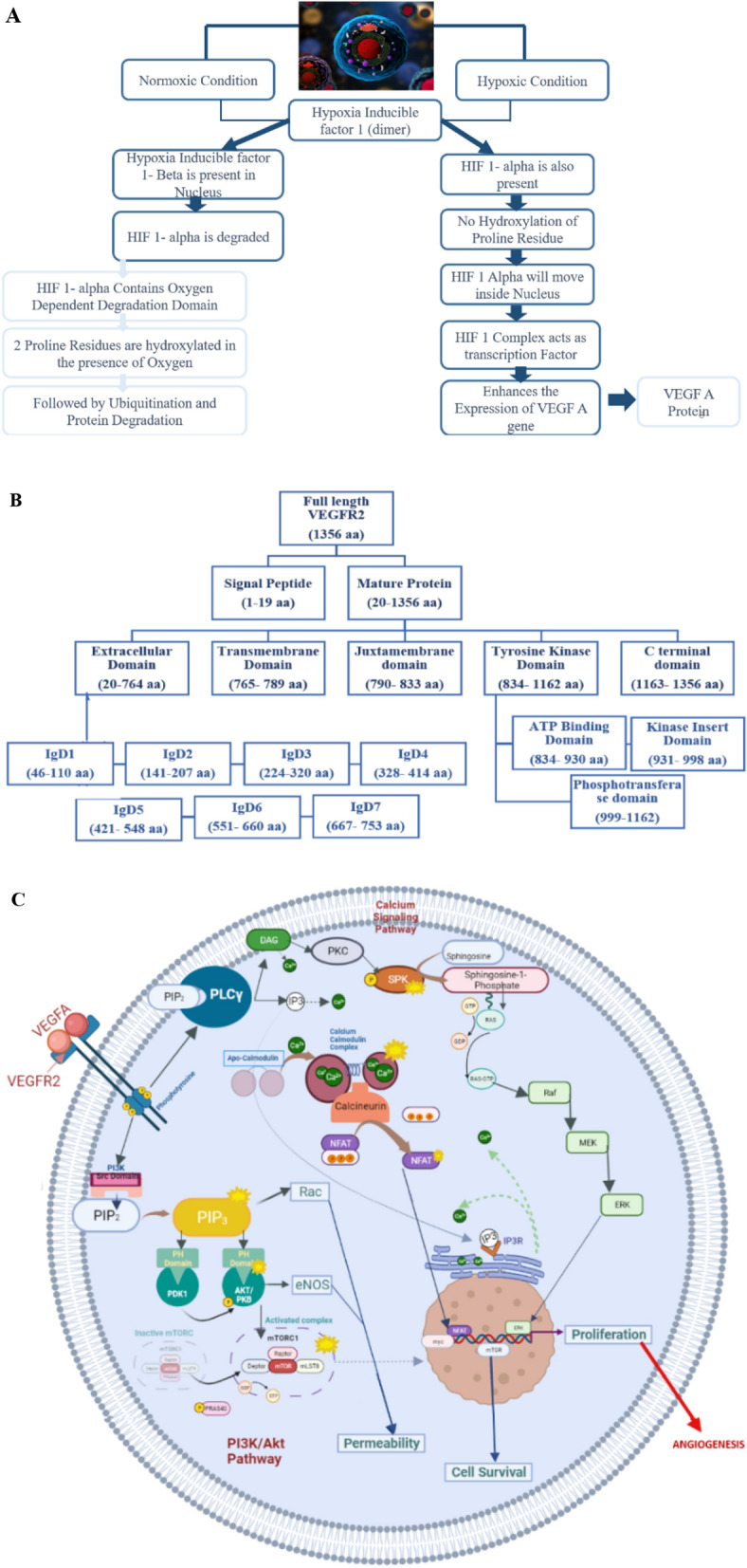
(**A**) Flow chart representing the comparison of Normoxic and hypoxic conditions in a cell that triggers activation of VEGFA protein^53^. (**B**) Flow chart representing the domains of VEGFR2^11^. (**C**) Diagrammatic representation of VEGFA-VEGFR2 downstream signaling pathways ^15,54^.

RA is an inflammatory autoimmune disease and it is one of the major reasons for disability^1^. RA is characterized by synovitis, damage to articular cartilage and several other bone issues^2,3^. RA accounts for 0.5–1% in general population^4^. 0.55–1.9% of population in Pakistan is affected by RA^5^. RA is 2 to 3 times more prevalent in females as compare to males^6^. Synovial angiogenesis is also observed in RA patients which is responsible for synovitis. The major proangiogenic factor is Vascular growth factor A that is released under hypoxic conditions by the cells which are deprived of oxygen to fulfil oxygen requirement^7^. Hypoxia inducible factors (HIF) are group of heterodimeric transcription factors which are the key regulators to show active response against varying oxygen conditions in body and angiogenesis is the major response against HIFs to fulfil the oxygen requirement. Figure 1A.

The Vascular Endothelial Growth factor (VEGF) is located on chromosome 6p12. It is comprised of 8 exons and 7 introns and the coding region spans around 14 kb^8^. The VEGF family is comprised of VEGF-A, VEGF-B, VEGF-C, VEGFD, VEGFE, VEGF-F and PLGF (placental growth factor). Genome sequencing of VEGF have confirmed that all the families of VEGF have 8 cysteine residues which are conserved at fixed position that are closely related to PDGF family^9^. VEGF-A is also known as VEGF^10^. VEGFA is a homodimer protein which is secreted by different types of cells like endothelial cells, smooth muscle cells, macrophages, fibroblasts, neutrophils, platelets and many tumor cells. VEGFA protein is formed by vegfa gene. This gene undergoes alternative splicing to give different isoforms of VEGFA protein. There are 6 different transcripts of VEGFA reported these transcripts generates 16 different isoforms. The reported transcripts of vegfa gene are VEGFA-111, VEGFA-121, VEGFA-145, VEGFA-165, VEGFA-189, VEGFA-206. VEGFA-165 was the first isoform which was identified and it is the most extensively studied isoform^11^. The transcript selected for this research is VEGFA-206. This transcript is the largest among all others. This transcript is composed of 232 amino acids^12^. In VEGFA-206 all the exons remained conserved from 1-8a except for exon 8b that is removed by alternative splicing hence it is considered as largest isoform^11^. VEGFA initiates the process of angiogenesis upon binding with VEGFR2.

Vascular Endothelial Growth factor receptor (VEGFR-2) is a Kinase Insert domain (KDR gene) receptor is transmembrane glycoprotein which is located on chromosome locus 4q11-12. It possesses 7 extracellular Ig-like domains (immunoglobulin like domains) one transmembrane helix and in the cytoplasm there is a split tyrosine kinase domain^11^. Immunoglobulin like sub domains including IgD2 and IgD3 (141–320 aa) are involved in tight binding of VEGFA dimer to VEGFR2 for its dimerization and activation^13^. Figure 1B is showing the domains of VEGFR2. The binding of VEGFA homodimer to VEGFR2 initiates PI3K-AKT pathway, Calcium Signaling Pathway and Calmodulin pathway which leads to cell proliferation and angiogenesis. Figure 1C is showing the downstream pathways activated upon binding of VEGFA with VEGFR2^14,15^.

VEGFA polymorphism has also been reported to have association with RA in different populations^16,17^. A single nucleotide polymorphism can affect the overall protein stability, functioning, pathogenicity, protein–protein interactions and protein nature. Vegfa is reported as highly polymorphic gene possessing high number of genetic polymorphisms i-e. 30 functional SNPs in promoter region, 5’UTR region, 3’ UTR region^18,19^. Due to polymorphic nature of VEGFA it may influence the expression of this gene between different individuals. According to different functional studies, VEGFA variants were directly associated with mRNA levels and the protein expression in patients with different angiogenic diseases^20^.

## Methodology

**Figure 2:**
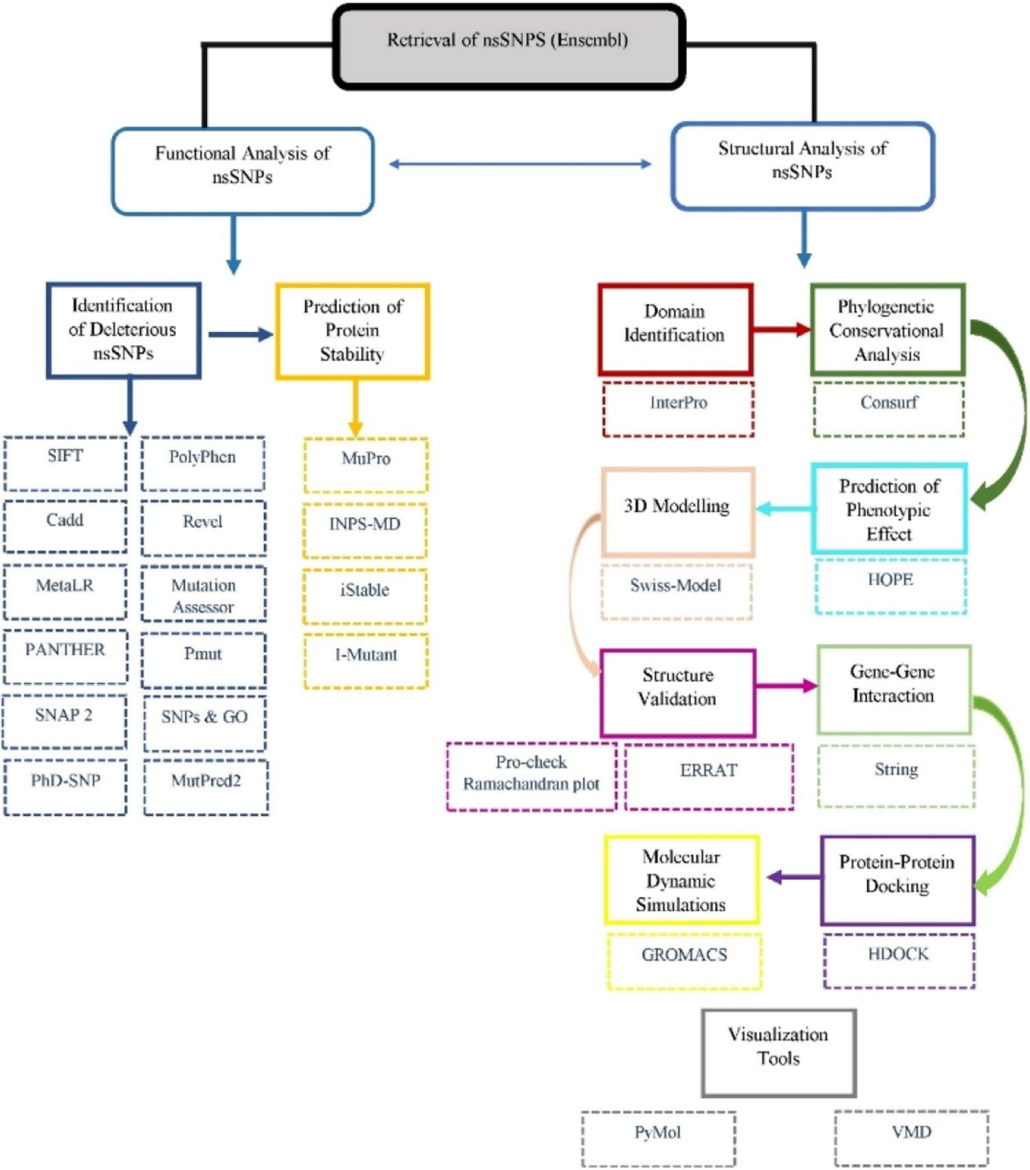
Flow chart showing the methodology performed for current research.

Figure 2 is showing flowchart of methodology performed in current study.

### Data retrieval

Primary Sequence and SNPs of VEGFA gene (*homosapians*) was retrieved from NCBI and Ensemble. All the SNPs were classified by Ensemble into 10 comprehensive groups depending upon their genomic location including splice acceptor variants, splice donor variants, non-synonymous variants, synonymous variants, splice polypyrimidine variants, splice region variants, 3′ UTR and 5′ UTR variants, coding sequence variants and intron variants.

### Mining of deleterious nsSNPS

To determine the pathogenic nature of nsSNPs, twelve webserver and tools were employed. All tools were used according to their defined protocol and default settings. We used SIFT^21–23^, Polyphen (Polymorphism Phenotyping)^24,25^, Cadd^26^, Revel^27^, Mutation assessor^28^, and MetaLR the meta predictor logistic regression (LR)^29^, After using these six web tools we neglected those mutations which were showing neutral effect and selected those nsSNPs which were observed with pathogenic effect. Further we used PHD-SNP, PANTHER, SNAP2^30,31^, SNPs and GO (single nucleotide Polymorphism Database & Gene Ontology)^32^, PMut^33^, MutPred 2^34^ to select highly pathogenic nsSNPS.

### Mining of nsSNPs in protein domains

Interpro server was used to classify and predict the functional domains of VEGFA protein. Protein’s FASTA sequence was subjected to the tool to identify the conserved domain present in VEGFA. After retrieving domains of our protein, the position of the selected nsSNPs were visualized via PyMOL^35^.

### Prediction of evolutionary conservation of VEGFA

The conservation profile of VEGFA protein was obtained using ConSurf server. This tool uses an empirical Baysian method to give the conservation scale of each residue. The conservation score ranges from 1 to 9 where the score ranging from 7 to 9 are considered as conserved residues^36^. Protein sequence of VEGFA was subjected for identifying the conserved residues involved in structure and function of VEGFA gene.

### Prediction of phenotypic effect

HOPE web server was used to analyze the effects of point mutation on the phenotype of VEGFA gene. HOPE finds homologs sequences from different databases by using BLAST algorithm and generates output by giving information about changes in protein structure, hydrophobicity, size of protein and electron density^37^.

### Effect on stability of a protein

To determine the stability, change of protein after an amino acid substitution have an important role either mutation/ substitution will have effect on configuration of protein or not by increasing or decreasing the protein stability. Pathological mutations and those substitutions that are specifically in the conserved region of a protein can affect the stability of a protein. In order to determine impact of nsSNPs on overall stability of VEGFA MUpro^38,39^ I-mutant 2.0^40^, INPS-MD^41^ and iStable^42^ were used by comparing on Gibbs free energy.

### VEGFA protein interaction by STRING database

The protein–protein interaction of VEGFA were identified by using STRING database. The database predicts both direct (physical) and indirect (functional) interactions. The server is linked to several other databases to provide in-depth and detailed map of protein–protein interaction^43^.

### Prediction of 3D structure-Swiss model

The homology modelling also known as Comparative modelling was performed by using Swiss-Model, which builds 3D structure of protein having known sequence but the structure is unknown. We superimposed wild-type model with all the mutants to visualize the tertiary confirmational change and root mean square deviation (RMSD). After successfully generating wild and mutant models they were saved in.pdb format for visualization using PyMOL.

### Structure validation

For further validation of protein models obtained from Swiss-Model Saves-Procheck server for construction of Ramachandran Plots and ERRAT was used. By Ramachandran Plot we can predict which secondary structure is associated with given amino acid of a protein^44,45^. ERRAT helps to determine overall model quality by giving a score percentage^46^.

### Molecular docking

In order to identify the Protein–Protein interaction, molecular docking was performed using HDOCK server^47^. VEGFA wild and mutants were docked against IgD2 and IgD3 domains of VEGFR2. The VEGFA molecules specifically binds to these domains of VEGFR2 and intracellular cascades are activated which triggers angiogenesis. So, the aim of this step was to check the impact of interactions of selected non-synonymous mutations of VEGFA protein with VEGFR2. The structure of IgD2 and IgD3 (KDR also known as VEGFR2) protein was retrieved from PDB and submitted to HDOCK to carry out binding interaction mechanism. Pymol was used for visualization of these docking complexes to check interactions of wild and mutants with VEGFR2^48^.

### Molecular dynamic simulations

MD Simulations were carried out by using GROMACS (GROningen MAchine for Chemical Simulations). Simulations were performed for 50 ns (ns) for all the mutants and wild type. After performing simulations, the resultant data was retrieved and analyzed including Root mean square deviation (RMSD), Solvent-Accessible surface area (SASA), Root mean square fluctuations (RMSF). MD simulations were performed to comprehend structural outcomes of all the mutants that affect VEGFA- IgD2 and IgD3 domains of VEGFR2 complexes interactions with respect to time. We predicted behavior and motion of protein in fourth dimension under principles of Newton’s laws. After that VMD^49^, a visualization program was used to analyze the trajectories.

## Results

### SNP annotation

**Figure 3:**
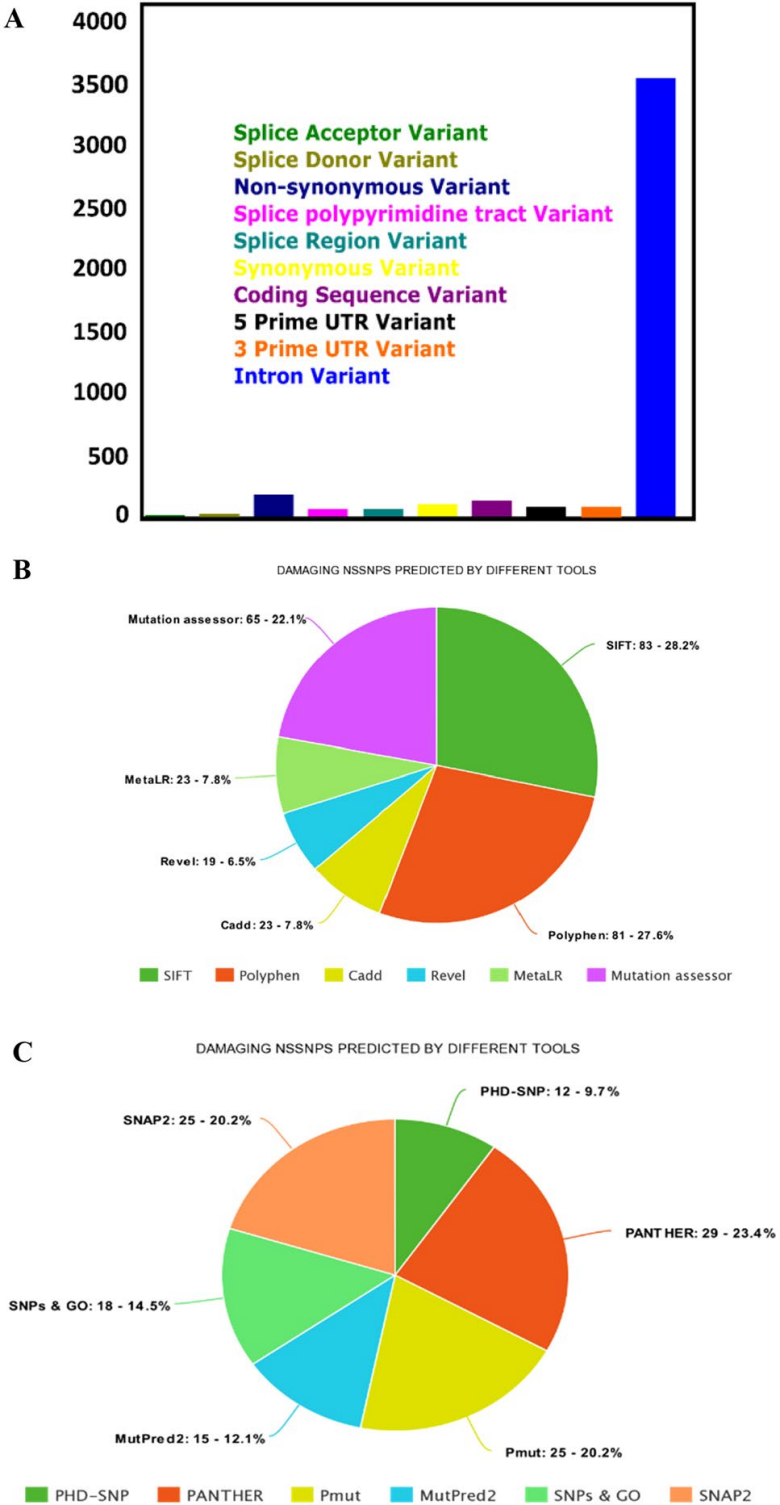
(**A**) Vertical Bar graph representing the annotation of VEGFA-206 transcript. (**B**) Pie chart showing percentages of mutants predicted by SIFT, Polyphen, Cadd, Revel, MetaLR, and Mutation Assessor. (**C**) Pie chart showing percentages of mutants predicted by PhD-SNP, Panther, Pmut, Mutpred2, SNPs &GO, and SNAP2.

Ensemble provided total 26 splice variants (transcripts) of VEGFA gene. VEGFA-206 was selected for this study. This transcript is based on 784 base pairs and consist of 232 amino acids. It comprises of 8 exons and 4,172 allelic variants. Missense/Non-synonymous variants were selected for further in silico analysis. Graph in Fig. 3A is showing annotation of VEGFA transcript.

### Predicted pathogenic mutations

All the non-synonymous variants were analyzed through 6 different computational tools to identify the pathogenic SNPs. The tools used for mining of nsSNPS were SIFT, Polyphen, CADD, Revel, MetaLR and Mutation Assessor. 168 nsSNPs of VEGFA were submitted to SIFT, out of them 83 nsSNPs were deleterious. The threshold Index for SIFT was 0.5. SNPs having score less than 0.5 were considered as damaging. Among the deleterious SNPs 40 were having score of 0 and were categorized as highly deleterious (Table S1). Polyphen predicted 81 missense mutations out of 168 with a score > 0.85 as damaging (Table S2). Cadd predicted 23 nsSNPs with score greater than 30 as deleterious (Table S3). Revel identified 19 mutations having confidence score greater than 0.5 and were considered as deleterious (Table S4). MetaLR predicted 23 non-synonymous mutations as damaging with the score greater than 0.5 (Table S5). According to results of mutation accessor, confidence score for having high probability of being deleterious was greater than 0.9 and the tool predicted only 2 mutations for having high chance of pathogenicity. 63 mutations out of 168 were having medium pathogenicity with confidence score ranging between 0.5 and 0.9 (Table S6). We selected 29 mutations/168 that were observed to be deleterious by SIFT as well as Polyphen. It was supporting the fact that all the results that are predicted on sequence basis by SIFT are in correlation with structure-based results obtained from Polyphen. On the other hand, there were few mutations that were predicted tolerated/ likely benign by CADD, Revel, MetaLR and Mutation Assessor but they were showing deleterious effect in SIFT and Polyphen. We highlighted them as well for further analysis by other mutation prediction tools. The consequences of above mentioned 6 tools (Fig. 3B) were further confirmed by 6 other mutation prediction tools including PHD-SNP, PANTHER, PMut, MutPred2, SNPs & GO and SNAP2 (Fig. 3C). According to the results of PHD-SNP server 12/29 mutations were showing disease causing effect. PANTHER predicted 24 nsSNPS as probably damaging and 5 mutations were showing possibly damaging effect, both were considered as deleterious. PMut classified only 4 missense mutations as neutral with confidence score < 0.5. MutPred2 identified 15/29 missense mutations showing pathogenic effect with score > 0.5. It also predicted the molecular mechanisms that are altered by addition of mutation given in Table 1. SNPs & GO predicted 18 missense mutations as disease causing. SNAP 2 generated the results in the form of heatmap (Fig. S1), which predicted 14 nSNPs as effective with score 0–50, and 11 were predicted as highly effective with SNAP score 50–100 (Table S7).

**Table 1:** Molecular mechanisms of mutations predicted by MutPred 2.

Substitution Position	Effect
V59M	Altered Transmembrane protein
V78M	Loss of Strand, Loss of Disulfide linkage at C77, Altered Metal binding, Altered Transmembrane protein
R82Q	Altered Metal binding, Loss of Disulfide linkage at C77, Altered Transmembrane protein, Gain of Catalytic site at C83
C86Y	Altered Metal binding, Gain of Disulfide linkage at C87, Altered Transmembrane protein, Loss of Loop, Gain of Catalytic site at C86
E90K	Altered Metal binding, Gain of Disulfide linkage at C87, Altered Transmembrane protein
R108W	Loss of Intrinsic disorder, Altered Transmembrane protein, Altered Stability
R155C	Loss of B-factor, Gain of Acetylation at K152
R155L	Loss of B-factor, Gain of Acetylation at K152
C204S	Loss of Disulfide linkage at C206
C204R	Loss of Disulfide linkage at C206
R216M	Gain of Loop, Gain of Disulfide linkage at C213
R223G	Gain of Loop
R223C	Gain of Loop, Altered Metal binding
R232W	Loss of B-factor, Altered Metal binding
R232Q	Gain of Pyrrolidone carboxylic acid at R232

After refining results from 12 different computational tools, we neglected those mutations which were resulting as benign in most of the tools. We highlighted those mutations which were sharing common results in maximum number of tools used for analysis of their pathogenicity. After manually screening method 19 nsSNPS were selected with high robustness for further study.

### Mining of nsSNPs in VEGFA domains

According to Interpro results VEGFA contains two domains including PDGF/VEGF domain (39–135 aa) and Heparin-binding domain (183–232 aa) as shown in Fig. S2. This domain play role in Heparin binding and it is present in C-terminus of VEGF. According to the results of InterPro all of our nsSNPs are present in the first domain. After analysis from InterPro server our missense mutations were divided into two domains PDGF/VEGF domain containing 9 missense mutations and Heparin-binding domain possessing 10 missense mutations.

### Evolutionary conservation analysis

**Figure 4:**
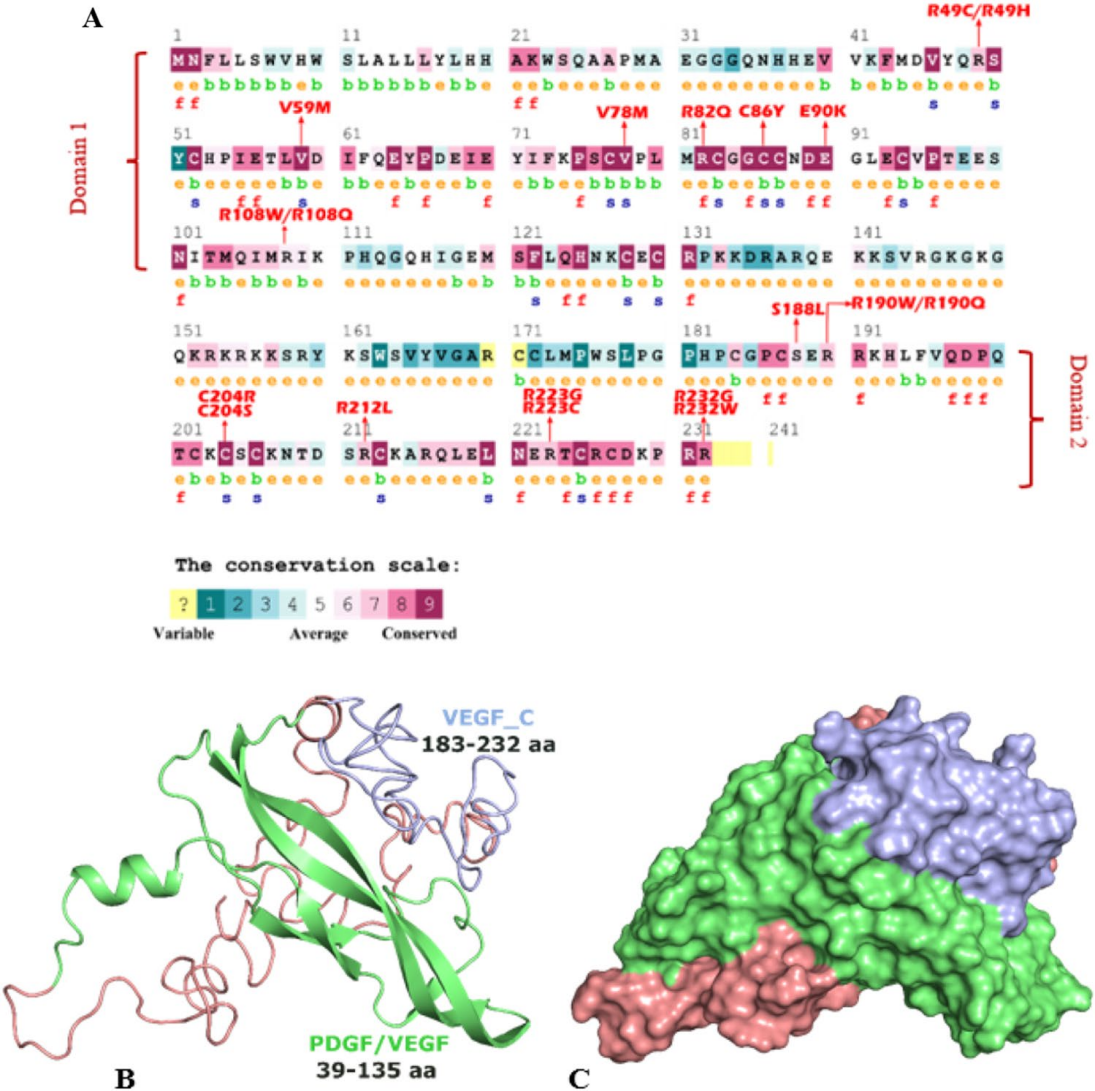
(**A**) Conservation profile of VEGFA Predicted by ConSurf. (**B**) Cartoon structure of VEGFA (C) Surface structure of VEGFA.

Protein residues which are highly conserved are more likely responsible for structural and functional integrity of the protein. ConSurf predicted the evolutionary conservation profile of VEGFA (Fig. 4A), according to the results 7 out of 9 SNP positions are more conserved with conservation score of 7–9 in the first domain. Among these 7 SNP positions 5 are highly conserved residues with score of 9. If amino acid residues are highly conserved it means they have more significant biological functions. According to the results of ConSurf 3 out of 9 positions were heavily buried in first domain which indicates that the mutation in these residues will affect the structural integrity of the protein. On the other hand, 6 residues were exposed and among those 6 residues only 2 residues (rs762664023, rs1421145908) showed functional affect which indicates that mutation in those 2 residues will affect the functioning of VEGFA protein. In the second domain of VEGFA 7 out of 10 residues are highly conserved with score 7–9. The conservation value of all the 19 SNPs along with their effect is given in supplementary Table S8.

According to the predictions of InterPro a loop between two domains confirmed that it possesses highly variable residues with lowest conservation score. Similarly, we selected 9 nsSNPS that would be of prime significance in VEGFA association analysis with Rheumatoid Arthritis. Furthermore, the first domain (PDGF/VEGF domain, 39–135 aa) opted conserved residues sited for missense mutations, other exposed residues with functional effect and buried residues with structural effect are located in the PDGF/VEGF domain., Both of the domains cannot be selected because variable region can form loop in structural analysis, for all these reasons we picked out 9 nsSNPs from the first domain for further study.

### Predicted protein stability

To predict the effect of all the deleterious nsSNPs on the stability of VEGFA protein four tools were used: MuPro, I-Mutant 2.0, INPS-MD and iStable. We analyzed stability change in VEGFA by free energy values. According to results of MuPro and iStable and INPS-MD, 8 out of 9 nsSNPs showed decrease in protein stability after mutation whereas only rs1208889729 showed increase in stability (Table 2). Prediction by I-mutant showed that 7 nsSNPS were affecting the protein by decreasing its stability and two nsSNPS (rs759253179, rs374420337) increased the protein stability. Altogether 8 nsSNPS that were reported as deleterious can destabilize the protein as well.

**Table 2:** Predicted stability change of all the mutants by MuPro, iStable, INPS-MD, I-mutant.

MuPro			iStable		INPS-MD		I-mutant	
Amino acid Substitution	Stability	Confidence Score	Stability	Confidence Score	Stability	DDG	Stability	DDG
R49C	Increase	0.2779	Increase	0.54929	Increase	0.18115	Decrease	−1.82
R49H	Decrease	−0.131	Decrease	0.847064	Decrease	−0.982838	Decrease	−2.89
V59M	Decrease	−0.967	Decrease	0.818099	Decrease	−0.834608	Decrease	−0.1
V78M	Decrease	−0.083	Decrease	0.768939	Decrease	−0.88301	Increase	0.34
R82Q	Decrease	−0.895	Decrease	0.745915	Decrease	−1.27834	Decrease	−0.57
C86Y	Decrease	−0.186	Decrease	0.780151	Decrease	−1.51311	Increase	0.16
E90K	Decrease	−0.278	Decrease	0.863849	Decrease	−0.571627	Decrease	−0.31
R108W	Decrease	−0.647	Decrease	0.861736	Decrease	−0.535469	Decrease	0
R108Q	Decrease	−1	Decrease	0.874011	Decrease	−0.773578	Decrease	−0.56

### Predicted phenotypic effect

Project HOPE was used to determine effects of nsSNPs of VEGFA on size, charge spatial structure and function of amino acid. Table S9 is showing the results of HOPE server. According to predicted results the size of 4 pathogenic nsSNPS (R49C, R49H, R82Q, R108Q) after mutation decreased as compare to wild type whereas size of 5 residues (V59M, V78M, C86Y, E90K, R108W) after mutation got increased. Hence, we can interpret from the results that change of single amino acid may affect overall configuration of a protein. The change in physiochemical properties can affect protein–protein interactions they can also affect the protein function. Some of the mutations (V78M, R82Q, C86Y) were located in close vicinity to the residue involved in cystine bridge formation hence mutation in these residues were affecting tertiary confirmation of protein. Besides this HOPE predicted V59M, V78M, and C86Y as buried residues hence when the wildtype got mutated, the new residues were unable to fit inside the protein core due to loss of interactions and larger size of mutated residues.

### Predicted tertiary structure

The full-length 3D structure of VEGFA is not available in PDB. The structure of PDGF/VEGF and VEGFC are present in PDB. They were used as a template for structural homology modelling of VEGFA. As per results of Interpro and Consurf most of our non-synonymous conserved mutations are present in the first domain (PDGF/VEGF) of VEGFA so structural analysis of nsSNPs present in this domain was carried out. The homologous model was generated using a template 5fv1.1.D. The model predicted by Swiss Model was comprising of residues from glu39- arg136 amino acids Fig. 4B,C. This model covered 98 residues out of total 232 residues of VEGFA protein. All the highly conserved, non-synonymous mutations were already part of this particular predicted structure. The QMEAN score for our wild-type model was 0.75 which suggested that our model is reliable.

### Regiospecificity of mutants

**Figure 5:**
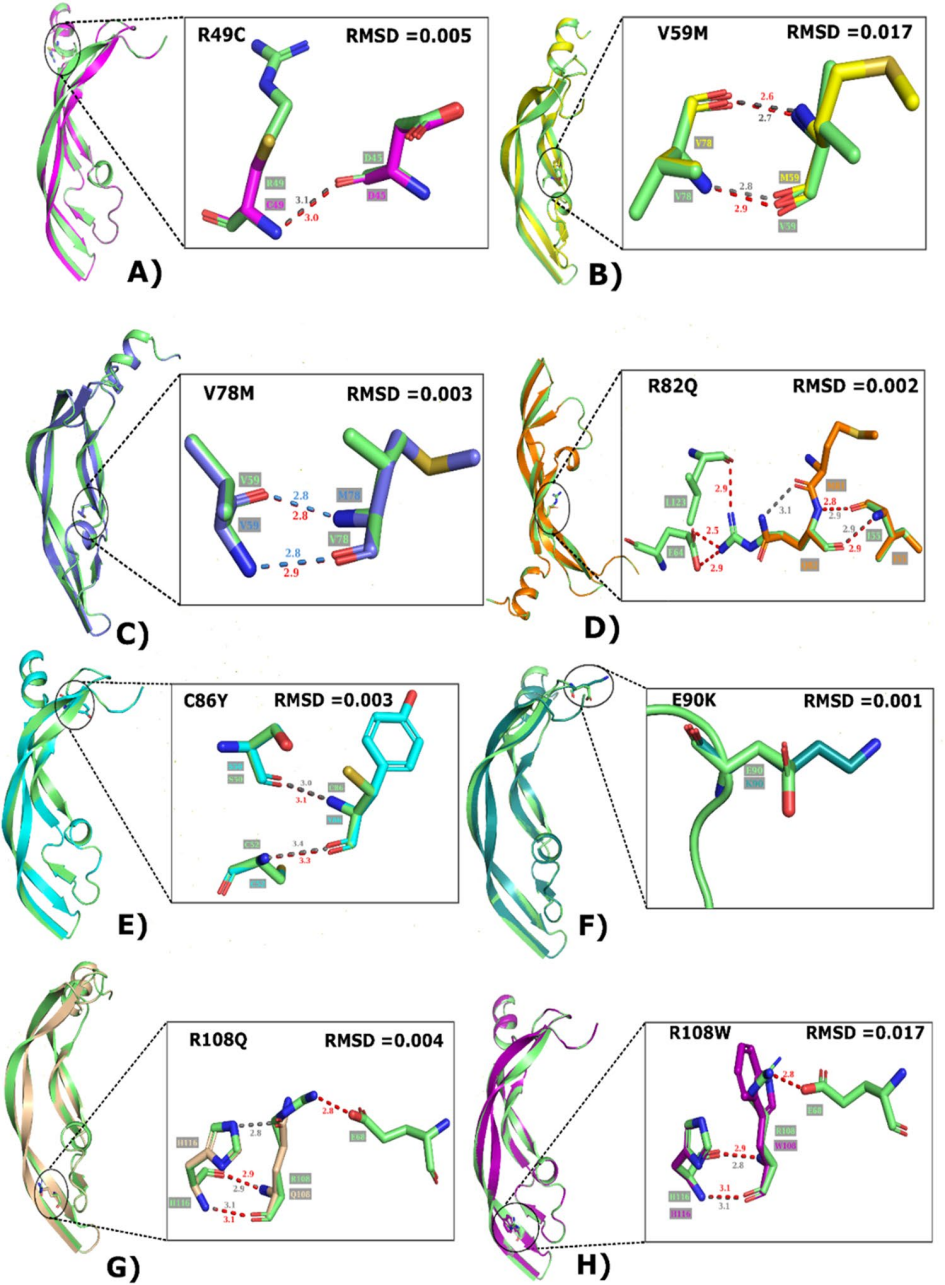
A Super-imposed structures of wild and mutants. (**A**) R49C with wild. (**B**) V59M with wild. (**C**) V78M with wild. (**D**) R82Q with wild. (**E**) C86Y with wild. (**F**) E90K with wild. (**G**) R108Q with wild. (**H**) R108W with wild.

After structural prediction of VEGFA (native and mutants) we further superimposed native VEGFA with all the mutants to confirm deviation of mutants from wild (Fig. 5). High RMSD value predicts higher deviation of mutant from wild and if RMSD value is less it means that there is less structural deviation after introduction of mutation. The mutant V59M and R108W showed highest structural deviation among others with RMSD of 0.017.

### Model validation

**Figure 6:**
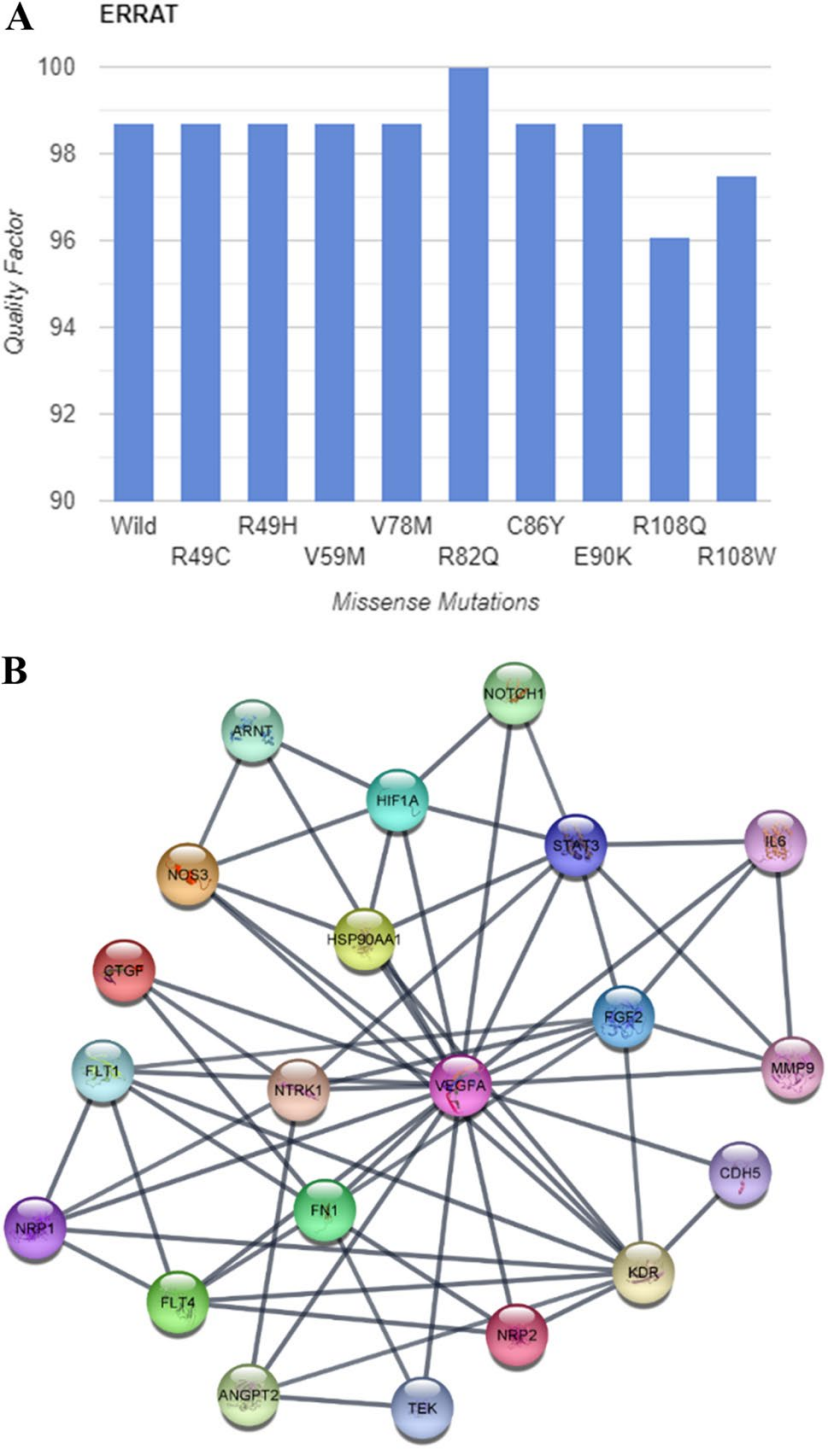
(**A**) Bar graph showing the quality factor of wild and all the mutants predicted by ERRAT. (**B**) Protein–Protein Interaction predicted by STRING database.

The quality of all the predicted models was verified by ERRAT which predicted that overall quality factor was more than 95% for wild and mutated models (Fig. 6A). Ramachandran plots were constructed using procheck to validate the structure of protein modelled from SWISS-Model (Fig. S3). According to the constructed plot, 92.9% residues are in favored region and 7.1% of the residues are in allowed regions. The favored region does not have any steric hinderance. The plot also predicted that most of the secondary structures are beta sheets. So, beta sheets are most prevalent in PDGF/VEGF domain of VEGFA protein.

### Protein–protein interaction

STRING was used to perform the protein–protein interaction (PPI) network analysis. According to the results VEGFA was interacting with 30 other proteins (Fig. 6B). Among these proteins the interaction score of VEGFA was highest with FLT1, FLT4, KDR, NRP1 AND NRP2 having interaction score of 0.999.

### Molecular docking analysis

**Figure 7:**
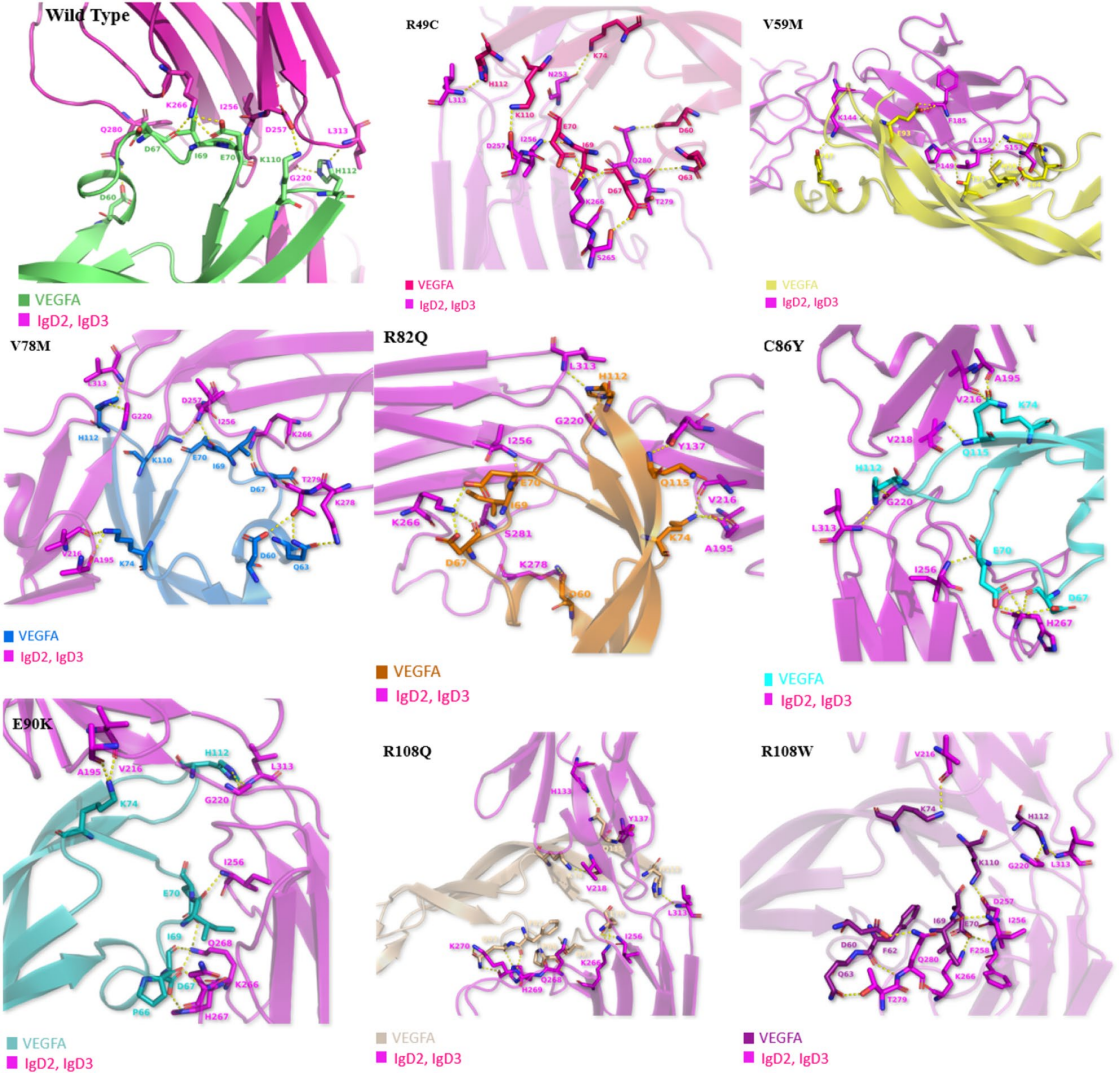
Docked complexes of Wild and Mutant VEGFA with IgD2 and IgD3 domains of VEGFR2.

Protein–Protein docking was performed using H-dock server. Comparative interaction analysis of native VEGFA and mutants docked with IgD2 and IgD3 domain of VEGFR2 (Fig. 7) showed some conserved and number of new polar contacts which are given in supplementary Tables S10 and S11. The docking score showed by Wild Type was -303.72, R49C -308.11, V59M -300.82, V78M -314.78, R82Q -298.33, C86Y -297.28, E90K -299.37, R108Q -346.11, R108W -296.85 in kJ/mol.

## Molecular dynamic simulation of VEGFA and its mutants

### Stability analysis

**Figure 8:**
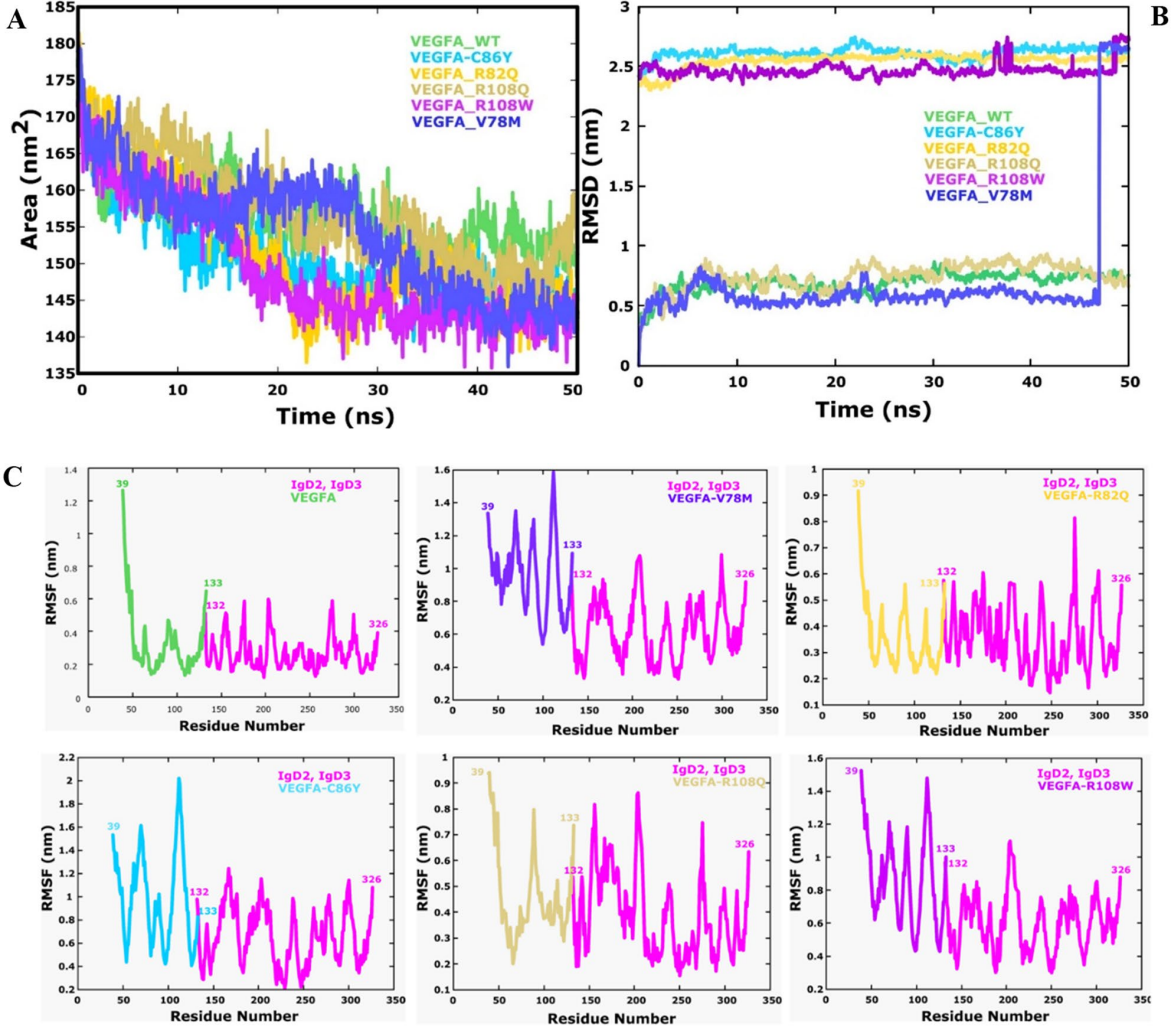
(**A**) Root mean square deviation (RMSD) of all the complexes. (**B**) SASA graph of wild and all the mutant complexes (**C**) Root mean square fluctuation (RMSF) of wild-type complexed with VEGFR2, V78M complexed with VEGFR2, R82Q complexed with VEGFR2, C86Y complexed with VEGFR2, R108Q complexed with VEGFR2 and R108W complexed with VEGFR2.

We performed stability analysis by calculating RMSD (Fig. 8A), which predicts that if deviation is high from native the structure will be unstable. So, stability analysis showed that wild and mutant R108Q were more stable throughout the simulation of 50 ns. The mean RMSD recorded for wild type is 0.5–1 nm throughout the time period of 50 ns simulation. Mutant R018Q showed same fluctuation as wild type and average RMSD for R108Q also remained between 0.5 and 1 nm. The mutant V78M also showed stability till 47 ns after that it lost its stability due to change in ligand pose and it showed gradual uphill accent till 2.6 nm where it became stable again after 48 ns. Other mutants including R82Q, R108W, C86Y showed similarity among themselves with respect to RMSD. The average RMSD recorded for these three mutants was 2.5 nm throughout the time of 50 ns. R108W showed a minor notch uphill after 38 ns with RMSD approximate 2.7 nm which again came back to mean RMSD at 40 ns. So, there were two mean RMSD recorded for wild and its mutants, three structures (native, R108Q, V78M) showed mean RMSD of approximately 0.7 nm and remaining three mutants (C86Y, R82Q, R108W) showed mean RMSD of approximately 2.6 nm. The two mutants V59M and E90K showed inconsistent behavior in the system throughout the simulation which indicated their instability hence both of them were ignored.

### Solvent accessible surface area

To explore the dimensional discrepancy caused by mutants we calculated Solvent accessible surface area (SASA) of the protein structures (Fig. 8B). According to results, Wild-type has SASA of approximately 170 nm initially which gradually declines to approximately 153 nm. Mutant R108Q starts approximately from 170 nm and gradually declines at approximately 155 nm. Mutant V78M showed SASA starting from approximately 179 nm, then it showed decline till 20 ns where it ascends slightly till 30 ns and after that it showed gradual decline with approximately 140 nm. With respect to wild type the surface area covered by R108Q is also ranging from 170 to 150 nm. Other mutants including C86Y, R82Q, and R108W showed more deviation as compare to wild type covering the area from 175 to 140 nm in 50 ns of simulation time period. According to the results wild type and R108Q are occupying less surface area as compare to other mutants which means protein only have that particular surface area to show deviation. Less surface area covered by protein shows less deviation which indicates that the protein is more stable.

### Flexibility analysis

Flexible nature of a protein allows it to behave accordingly in the environmental changes. Protein’s flexibility regulates different functions such as regulating the protein activity, Enzymatic activity. So, if protein’s flexibility is disrupted it affects the function of particular protein^50^. To understand the flexibility changes of individual residues that are incorporated due to point mutation on VEGFA, we calculated RMSF. The fluctuation distribution of all the mutants and native structure interacting with VEGFR2 are shown in the Fig. 8C. RMSF analysis indicated that all the residues present in N terminus showed mean fluctuation of 1.3 Å except for V59M which showed fluctuation of 3.6 Å.

RMSF analysis for C86Y with respect to wild type showed that residues in N terminal regions have almost same fluctuation till 50^th^ residue. After 50^th^ residue there is change in behavior of C86Y mutant. From 50 to 80 residue in mutant, showed high fluctuation with the highest one showing fluctuation of 1.6 Å. It was observed that residues in 100–140 position constituted a comparatively highest flexible region (2 Å) as compare to wild type. The mutant E90K also showed similar patterns of fluctuation, residues from 50 to 80 showed a flexibility in fluctuation (2.4 Å). The residues between 80 and 100 were comparatively more stabilizing with mean fluctuation of 1 Å. After 100 residue the RMSF peak got increased to 3 Å till almost 120^th^ residue which indicated that residues after 100 were highly unstable. By the introduction of mutation, the mutant C86Y and E90K has lost its stability as compare to wild type. The mutant R82Q was showing less fluctuation as compare to former two mutants. The residues from 50 to 80 showed highest RMSF of approximately 0.5 Å which was closely related to the wild type. The introduction of point mutation (Val78Met) changed the flexibility of residues from 0.1 A˙ to 0.8 A˙ respectively. There was not a significant change in flexibility of mutant but it effected other residues to change their flexibility. All the residues after insertion of Met at position 78 showed increase in fluctuation which means that stability of all the residues hence the whole complex decreased. The highest RMSF was recorded from residue 100–120 of 1.6 Å. The mutant R108Q showed decrease in RMSF from residual position 50–60 with minimum RMSF recorded 0.2 Å which was similar to wild type. The highest fluctuation was recorded from 70 to 100 residue was 0.8 Å. The residues in the mutant R108W also depicted high fluctuations as compare to wild type which indicated that this mutation also have decreased the overall stability of complex. The mutant V59M and E90K showed abnormally high RMSF which indicated that the complex is highly destabilizing hence they were ignored. The RMSF of R108Q recorded in 50 ns time period showed that fluctuation of residues was almost similar to wild type. The RMSF of all the mutants and wild type at different peaks are given in supplementary tables (Table S12, S13, S14, S15, S16, S17, S18).

## Discussion

RA is chronic disorder that is characterized by inflammation causing progressive damage to joint, pannus formation resulting in destruction of articular cartilage, synovial hyperplasia and synovial angiogenesis. RA is placed in a group of “angiogenic family of diseases” as it involves tissue neovascularization. VEGFA is considered as most potent angiogenic factor, as it is involved in formation of new blood vessels under the hypoxic condition. In the present study we have investigated about the VEGFA polymorphisms. A total of 4,172 allelic variants of VEGFA were obtained from Ensemble among them 168 were missense variants. For validation of results different tools must be used because each tool has a sophisticated algorithm so by using different tools, we can compare data from each tool and conclude the results. So, after analyzing each mutation in 12 different computational tools we ended-up with 19 highly pathogenic non-synonymous SNPs which were showing deleterious effect with highest confidence score in maximum tools.

We identified the domains of VEGFA using Interpro server we concluded that VEGFA is divided into two domains PDGF/VEGF domain which start from 39th amino acid and ends at 135th and Heparin-binding domain that range from 183rd to 232nd amino acid. Between these two domains (136–182 aa) there was unidentified loop region. The template for this region is not available on PBD hence the structure from amino acid 136 to 182 of VEGFA is unpredicted. Hence, we were aimed to select one particular domain because loop region can cause hindrance in further structural and docking analysis. Evolutionary conservational analysis using Consurf predicted that most of the conserved amino acids having highest conservation score were present in first domain so we selected 9 mutations present in PDGF/VEGF domain for further analysis. From stability analysis of VEGFA mutants by MuPro, I-Mutant 2.0, INPS-MD and iStable we predicted that overall protein stability was decreased by all the mutations except for the rs1208889729. So, decrease in protein stability refers to the phenomena that all the mutations were destabilizing which means that after introduction of point mutation the stability of VEGFA was decreased. HOPE server predicted change in phenotype of VEGFA after each mutation. According to results of HOPE server, all the mutations were causing drastic change on overall protein’s structure. The model generated by Swiss-model was covering residues from glu39- arg136 which means that Swiss-Model generated the 3D structure of only first domain. A total of 98 residues were covered by this protein model. QMEAN score of our structure was 0.75 which confirms the reliability of our model for further analysis. We further generated the models of all the mutants as well. In order to check the change in structure after mutation we superimposed wild type with all the models and visualized them in PyMol. We calculated Root Mean square Deviation (RMSD) which tells how much our mutant structure is deviated from the wild type The higher RMSD value means there is high deviation of mutant alpha Carbon backbone from its wild-type. If the RMSD value is minimum it means that mutated structure is almost similar to its wild type and there is not much impact of mutation on structural chemistry of protein^51^. According to the results, the highest deviation was showed by V59M and R108W with RMSD 0.017. Whereas least deviation from wild-type was showed by V78M, R82Q, C86Y, E90K which indicates that polymorphism of VEGFA at these particular positions was having less impact on protein’s structure. For validation of our models, we constructed Ramachandran plot for both native and mutants. According to constructed plots, 92.9% of the residues were lying in most favorable region and remaining 7.1% residues were present in allowed region. A best model predicted by Ramachandran plot is having at least 90% residues in favored region. For further confirmation we used ERRAT server to check model quality factor, which gave quality percentage of all the models greater than 95%. If the score is greater than 95% it means the protein model is validated by ERRAT and we can perform further analysis on that model^52^. We checked the interaction of VEGFA with other proteins by using STRING database, according to the results VEGFA was interacting with 30 other proteins. The highest interaction score was showed with FLT1, FLT4, KDR, NRP1 AND NRP2. Among these genes KDR was selected for docking analysis because KDR encodes Vascular Endothelial Growth Factor Receptor 2 (VEGFR2) on which our VEGFA molecule actively bind to initiate downstream pathways to trigger angiogenesis. We performed protein–protein docking using H-Dock server. VEGFA wild and mutants were docked on the IgD2 and IgD3 of VEGFR2. According to the results only few polar contacts remained conserved in all the mutants with respect to wild type. Number of new interactions among mutants with VEGFR2 were observed that were actually not present in wild type.

In order to validate our docked models, we performed Molecular Dynamics Simulations to check the docked interactions in dynamic environment. The simulation was performed using Gromacs for 50 ns. We determined RMSD, RMSF, and SASA for wild and mutants. According to the our findings mutant R108Q was showing RMSD pattern similar to wild type which suggest that this mutant was showing behavior almost similar to wild type. This mutant also showed less RMSF in comparision to other mutants. The least RMSF means that the mutant is highly stable. The SASA analysis depicted that mutant V78M and R108Q less deviation pattern similar to wild type as compare to other mutants which confirms their stabilizing nature.

This study predicted high-risk SNPS in VEGFA. We confirmed by docking and simulations how these mutations can disrupt the interactions of VEGFA with VEGFR2. However, there is need to perform invitro study on these mutations in different populations to check their presence in patients with RA.

## Conclusion

Current study concluded that, after performing screening analysis using different bioinformatics approaches, we got 2 nsSNPS of VEGFA that can be damaging and these mutations can affect the structure and/ or function of VEGFA. The in-depth analysis revealed the significance of mutations in PDGF/VEGF domain which can disrupt the interaction of this particular domain with the second domain. These nsSNPs can be further studied for purpose of therapeutic strategies and development of personalized medicines for patients suffering from RA. Among all the mutants the higher binding energy of R108Q with VEGFR2 showed that this complex was significantly more stable which confirms that if this mutation is present in a population, it can be more dangerous. As higher binding of VEGFA with VEGFR2 is directly related to high angiogenic rate, so presence of wild type and mutant R108Q can cause angiogenesis at high rate because there is need to identify drugs or biologics which can disrupt the binding of wild and mutant R108Q with VEGFR2 and act as anti-VEGF. So, biologics which can bind with VEGFR2 with similar binding energy as wild type and mutant R108Q, can slow down/ stop the angiogenic process and ultimately reducing pathogenesis of RA. Furthermore, functional analysis of these mutations is required to understand biological mechanisms of these polymorphisms in pathogenesis of RA, therefore it is needed to interpret downstream effects of all these pathogenic mutants on cellular signaling pathway and other biological processes. Protein expression, cell culture studies and enzymatic assays can provide more insight about the pathogenicity of the filtered mutants and their role in RA. There is a need to perform invitro population studies for these nsSNPs to check their prevalence in Pakistani population and their association with Rheumatoid arthritis.

### Supplementary Information

Supplementary Information.

## Data Availability

The data which is analyzed/ used under current research is available on request from the corresponding author.

## References

[1] Gary S. Firestein, Evolving concepts of rheumatoid arthritis. *Nature* 423: 356–361 (2003). Available: www.nature.com/nature. 10.1038/nature01661 12748655

[2] O’Shea JJ, Laurence A, McInnes IB (2013). Back to the future: oral targeted therapy for RA and other autoimmune diseases. Nat. Rev. Rheumatol..

[3] Akhtar M (2021). Characterization of rheumatoid arthritis risk-associated snps and identification of novel therapeutic sites using an in-silico approach. Biology (Basel).

[4] Carbonell J, Cobo T, Balsa A, Descalzo MÁ, Carmona L (2008). The incidence of rheumatoid arthritis in Spain: Results from a nationwide primary care registry. Rheumatology.

[5] Imran MY, Khan SEA, Ahmad NM, Raja SF, Saeed MA, Haider II (2015). Depression in rheumatoid arthritis and its relation to disease activity. Pakistan J. Med. Sci..

[6] Alam SM (2011). Epidemiology of Rheumatoid Arthritis in a tertiary care unit, Karachi, Pakistan. J. Pak. Med. Assoc..

[7] Miao CG (2013). Wnt signaling pathway in rheumatoid arthritis, with special emphasis on the different roles in synovial inflammation and bone remodeling. Cell. Signal..

[8] Hussain N, Sher SF, Lin X, Adil M (2022). Association of VEGF gene polymorphism (rs699947) with glaucoma and in-silico study of antiglaucoma bioactive compounds. Appl. Biochem. Biotechnol..

[9] Shibuya M (2011). Vascular Endothelial Growth Factor (VEGF) and Its Receptor (VEGFR) signaling in angiogenesis: A crucial target for anti- and pro-angiogenic therapies. Genes Cancer.

[10] Bakry RM, Hassan SM, Mohammed RA, Ablelaleem EA (2022). Significance of vascular endothelial growth factor (VEGFA)-1154 G/A gene polymorphism (rs1570360) in rheumatoid arthritis patients. Egypt. Rheumatol..

[11] Peach CJ (2018). Molecular pharmacology of VEGF-A isoforms: Binding and signalling at VEGFR2. Int. J. Mol. Sci..

[12] Houck KA, Ferrara N, Winer J, Cachianes G, Li B, Leung DW (1991). The vascular endothelial growth factor family: Identification of a fourth molecular species and characterization of alternative splicing of rna. Mol. Endocrinol..

[13] Wang X, Bove AM, Simone G, Ma B (2020). Molecular bases of VEGFR-2-mediated physiological function and pathological role. Front. Cell Dev. Biol..

[14] Cross MJ, Dixelius J, Matsumoto T, Claesson-Welsh L (2003). VEGF-receptor signal transduction. Trends Biochem. Sci..

[15] Takahashi H, Shibuya M (2005). The vascular endothelial growth factor (VEGF)/VEGF receptor system and its role under physiological and pathological conditions. Clin. Sci..

[16] Paradowska-Gorycka A (2016). Relationship between VEGF gene polymorphisms and serum VEGF protein levels in patients with rheumatoid arthritis. PLoS One.

[17] Chen Y, Dawes PT, Mattey DL (2012). Polymorphism in the vascular endothelial growth factor A (VEGFA) gene is associated with serum VEGF-A level and disease activity in rheumatoid arthritis: differential effect of cigarette smoking. Cytokine.

[18] Kim YJ, Chung WC, Jun KH, Chin HM (2019). Genetic polymorphisms of vascular endothelial growth factor (VEGF) associated with gastric cancer recurrence after curative resection with adjuvant chemotherapy. BMC Cancer.

[19] Aziz MA, Uddin MS, Millat MS, Islam MS (2022). Vascular endothelial growth factor A (VEGFA) promoter rs2010963 polymorphism and cancer risk: An updated meta-analysis and trial sequential analysis. Meta Gene.

[20] Wang Y (2017). Vascular endothelial growth factor A polymorphisms are associated with increased risk of coronary heart disease: A meta-analysis. Oncotarget.

[21] Ng PC, Henikoff S (2003). SIFT: Predicting amino acid changes that affect protein function. Nucleic Acids Res..

[22] Irfan M, Iqbal T, Hashmi S, Ghani U, Bhatti A (2022). Insilico prediction and functional analysis of nonsynonymous SNPs in human CTLA4 gene. Sci. Rep..

[23] Rajasekaran R, Sudandiradoss C, Doss CGP, Sethumadhavan R (2007). Identification and in silico analysis of functional SNPs of the BRCA1 gene. Genomics.

[24] Protoc, C. & Genet, H., *HHS Public Access* 2 (2015).

[25] Zhang M, Huang C, Wang Z, Lv H, Li X (2020). In silico analysis of non-synonymous single nucleotide polymorphisms (nsSNPs) in the human GJA3 gene associated with congenital cataract. BMC Mol. Cell Biol..

[26] Rentzsch P, Witten D, Cooper GM, Shendure J, Kircher M (2019). CADD: predicting the deleteriousness of variants throughout the human genome. Nucleic Acids Res..

[27] Ioannidis NM (2016). Article Revel: An ensemble method for predicting the pathogenicity of rare missense variants. Am. J. Hum. Genet..

[28] “Mutation assessor | NGRL Manchester.” http://www.ngrl.org.uk/Manchester/page/mutation-assessor.html (accessed May 09, 2023).

[29] Adzhubei IA (2010). A method and server for predicting damaging missense mutations. Nat. Methods.

[30] “Snap2 - Rost Lab Open.” https://rostlab.org/owiki/index.php/Snap2 (accessed May 09, 2023).

[31] Hecht M, Bromberg Y, Rost B (2015). Better prediction of functional effects for sequence variants. BMC Genom..

[32] Capriotti E, Calabrese R, Fariselli P, Martelli PL, Altman RB, Casadio R (2013). “WS-SNPs&GO: a web server for predicting the deleterious effect of human protein variants using functional annotation. BMC Genom..

[33] López-Ferrando V, Gazzo A, De La Cruz X, Orozco M, Gelpí JL (2017). PMut: A web-based tool for the annotation of pathological variants on proteins, 2017 update. Nucleic Acids Res..

[34] Pejaver V (2020). Inferring the molecular and phenotypic impact of amino acid variants with MutPred2. Nat. Commun..

[35] Paysan-Lafosse T (2023). InterPro in 2022. Nucleic Acids Res..

[36] Ashkenazy H (2016). ConSurf 2016: an improved methodology to estimate and visualize evolutionary conservation in macromolecules. Nucleic Acids Res..

[37] Venselaar H, Te Beek TA, Kuipers RK, Hekkelman ML, Vriend G (2010). Protein structure analysis of mutations causing inheritable diseases. An e-Science approach with life scientist friendly interfaces. BMC Bioinf..

[38] Cheng J, Randall AZ, Sweredoski MJ, Baldi P (2005). Scratch: A protein structure and structural feature prediction server. Nucleic Acids Res..

[39] Cheng J, Randall A, Baldi P (2006). Prediction of protein stability changes for single-site mutations using support vector machines. Proteins Struct. Funct. Genet..

[40] Capriotti E, Fariselli P, Casadio R (2005). I-Mutant2.0: Predicting stability changes upon mutation from the protein sequence or structure. Nucleic Acids Res..

[41] Savojardo C, Fariselli P, Martelli PL, Casadio R (2016). INPS-MD: A web server to predict stability of protein variants from sequence and structure. Bioinformatics.

[42] Chen CW, Lin J, Chu YW (2013). iStable: Off-the-shelf predictor integration for predicting protein stability changes. BMC Bioinf..

[43] Szklarczyk D (2019). STRING v11: Protein-protein association networks with increased coverage, supporting functional discovery in genome-wide experimental datasets. Nucleic Acids Res..

[44] Laskowski RA, MacArthur MW, Moss DS, Thornton JM (1993). PROCHECK: a program to check the stereochemical quality of protein structures. J. Appl. Crystallogr..

[45] Laskowski RA, Rullmann JAC, MacArthur MW, Kaptein R, Thornton JM (1996). AQUA and PROCHECK-NMR: programs for checking the quality of protein structures solved by NMR. J. Biomol. NMR.

[46] Colovos C, Yeates TO (1993). Verification of protein structures: patterns of nonbonded atomic interactions. Protein Sci..

[47] Yan Y, Tao H, He J, Huang SY (2020). The HDOCK server for integrated protein–protein docking. Nat. Protoc..

[48] Rigsby RE, Parker AB (2016). Using the PyMOL application to reinforce visual understanding of protein structure. Biochem. Mol. Biol. Educ..

[49] Humphrey W, Dalke A, Schulten K (1996). VMD: Visual molecular dynamics. J. Mol. Gr..

[50] Krami AM, Roky R, Barakat A, Nahili H (2019). Computational analysis of damaging nsSNP in human STXBP1 gene involved in early infantile epileptic encephalopathy: Molecular modelling and dynamics study. IBRO Rep..

[51] Schwede T, Kopp J, Guex N, Peitsch MC (2003). SWISS-MODEL: An automated protein homology-modeling server. Nucleic Acids Res..

[52] Agnihotry S, Pathak RK, Singh DB, Tiwari A, Hussain I (2021). Protein structure prediction. Bioinforma. Methods Appl..

[53] Konisti S, Kiriakidis S, Paleolog EM (2012). Hypoxia-a key regulator of angiogenesis and inflammation in rheumatoid arthritis. Nat. Rev. Rheumatol..

[54] Zachary I (2003). VEGF signalling: Integration and multi-tasking in endothelial cell biology. Biochem. Soc. Trans..

