# Supplementary material for: Association analysis of Vascular Endothelial Growth Factor-A (VEGF-A) polymorphism in rheumatoid arthritis using computational approaches
Source: Sci Rep. 2023 Dec 11;13:21957. doi: 10.1038/s41598-023-47780-8 (PMC10713577; doi:10.1038/s41598-023-47780-8)
Supplement: Supplementary file 1 — Supplementary Information. [file 41598_2023_47780_MOESM1_ESM.pdf]

Table S 1

Results predicted by SIFT

Accession	AA	AA coord	Prediction	Score	Accession	AA	AA coord	Prediction	Score
rs751447901	F/L	3	deleterious	0.03	rs1412676860	N/H	126	deleterious	0.01
rs757103787	W/R	7	deleterious	0.01	rs1412676860	N/D	126	deleterious	0.01
rs964195968	L/R	12	deleterious	0	rs1166395511	R/K	131	deleterious	0.04
rs1355739548	A/T	13	deleterious	0.04	rs76869573	P/T	132	deleterious	0.01
rs755797583	L/F	16	deleterious	0.03	rs760891966	E/V	140	deleterious	0.01
rs748846771	Y/N	17	deleterious	0	rs766541343	K/R	141	deleterious	0.04
rs1201834176	P/T	28	deleterious	0.02	rs1395807781	R/L	145	deleterious	0
rs138919899	N/I	36	deleterious	0.02	rs1475211079	K/T	152	deleterious	0.01
rs1193943585	E/K	39	deleterious	0.02	rs371177206	R/C	155	deleterious	0
rs763677053	V/A	40	deleterious	0.01	rs774265827	R/H	155	deleterious	0.03
rs1357683782	F/I	43	deleterious	0.01	rs774265827	R/L	155	deleterious	0.04
rs1005693041	V/I	46	deleterious	0	rs750127977	K/I	157	deleterious	0.03
rs1208889729	R/C	49	deleterious	0	rs141138308	R/W	159	deleterious	0.01
rs1267535717	R/H	49	deleterious	0.01	rs62401172	G/V	185	deleterious	0.03
rs1582497910	T/N	57	deleterious	0	rs1003930162	S/L	188	deleterious	0
rs755307045	V/M	59	deleterious	0	rs777632185	R/W	190	deleterious	0
rs748352475	P/A	66	deleterious	0	rs1429874344	R/Q	190	deleterious	0
rs772184987	P/L	66	deleterious	0	rs1465046427	K/M	192	deleterious	0
rs777797068	I/F	69	deleterious	0	rs1188254133	L/V	194	deleterious	0
rs933893718	E/K	70	deleterious	0	rs376388064	P/L	199	deleterious	0.03
rs910906787	I/F	72	deleterious	0.01	rs776522854	T/M	201	deleterious	0

**Accession**	**AA**	**AA coord**	**Prediction**	**Score**	**Accession**	**AA**	**AA coord**	**Prediction**	**Score**
rs770795261	I/M	72	deleterious	0.02	rs1287276985	C/S	204	deleterious	0
rs759253179	V/M	78	deleterious	0	rs1287276985	C/R	204	deleterious	0
rs762664023	R/Q	82	deleterious	0	rs1255510121	C/Y	204	deleterious	0
rs368256497	G/R	84	deleterious	0	rs369593555	R/L	212	deleterious	0.02
rs368256497	G/W	84	deleterious	0	rs866329410	A/V	215	deleterious	0
rs374420337	C/Y	86	deleterious	0.02	rs1189544809	R/M	216	deleterious	0
rs368814156	N/D	88	deleterious	0.02	rs749491856	N/H	221	deleterious	0
rs1421145908	E/K	90	deleterious	0	rs759826070	E/Q	222	deleterious	0.04
rs1459669662	E/G	93	deleterious	0	rs765514291	E/D	222	deleterious	0.02
rs1187507161	T/A	97	deleterious	0.02	rs752907384	R/G	223	deleterious	0
rs755361036	T/S	97	deleterious	0.02	rs752907384	R/C	223	deleterious	0
rs1346131223	T/A	103	deleterious	0	rs748984440	D/A	228	deleterious	0
rs114262569	R/W	108	deleterious	0	rs1272402969	P/A	230	deleterious	0
rs367757959	R/Q	108	deleterious	0.02	rs768366559	P/L	230	deleterious	0.02
rs1329473200	Q/R	124	deleterious	0	rs771561387	R/W	232	deleterious	0
rs771561387	R/G	232	deleterious	0	rs374253522	R/Q	232	deleterious	0
rs771561387	R/W	232	deleterious	0	rs374253522	R/Q	232	deleterious	0
rs374253522	R/Q	232	deleterious	0	rs374253522	R/Q	232	deleterious	0

Table S 2

Results predicted by Polyphen

Accession	AA	AA coord	Prediction	Score	Accession	AA	AA coord	Prediction	Score
rs751447901	F/L	3	probably damaging	0.938	rs763031344	K/E	154	probably damaging	0.991
rs757103787	W/R	7	probably damaging	0.932	rs371177206	R/C	155	probably damaging	0.997
rs964195968	L/R	12	possibly damaging	0.477	rs774265827	R/H	155	probably damaging	0.996
rs1355739548	A/T	13	possibly damaging	0.584	rs774265827	R/L	155	probably damaging	0.99
rs755797583	L/F	16	probably damaging	0.909	rs750127977	K/I	157	possibly damaging	0.834
rs375450405	Y/C	17	possibly damaging	0.551	rs267601048	S/F	158	possibly damaging	0.811
rs1201834176	P/T	28	probably damaging	0.987	rs141138308	R/W	159	probably damaging	0.997
rs1357683782	F/I	43	probably damaging	0.945	rs150806902	R/Q	159	probably damaging	0.99
rs1208889729	R/C	49	probably damaging	0.972	rs754475500	Y/C	160	possibly damaging	0.868
rs1267535717	R/H	49	probably damaging	0.972	rs769932323	Y/N	166	possibly damaging	0.865
rs375773218	I/M	55	possibly damaging	0.893	rs753526479	L/P	178	possibly damaging	0.906
rs1582497910	T/N	57	possibly damaging	0.869	rs139206878	P/S	179	possibly damaging	0.862
rs755307045	V/M	59	probably damaging	0.987	rs1345383477	P/L	179	probably damaging	0.912
rs748352475	P/A	66	probably damaging	1	rs1475279373	G/R	185	possibly damaging	0.472

rs772184987	P/L	66	probably damaging	1	rs62401172	G/V	185	possibly damaging	0.633
rs777797068	I/F	69	possibly damaging	0.565	rs1003930162	S/L	188	possibly damaging	0.812
rs933893718	E/K	70	probably damaging	0.981	rs777632185	R/W	190	probably damaging	0.959
rs910906787	I/F	72	probably damaging	0.914	rs1429874344	R/Q	190	possibly damaging	0.531
rs770795261	I/M	72	probably damaging	0.987	rs1465046427	K/M	192	probably damaging	0.999
rs1359856307	K/Q	74	possibly damaging	0.476	rs1176966214	H/R	193	probably damaging	0.998
rs759253179	V/M	78	probably damaging	0.985	rs1188254133	L/V	194	probably damaging	0.997
rs762664023	R/Q	82	probably damaging	0.997	rs776522854	T/M	201	probably damaging	0.999
rs368256497	G/R	84	possibly damaging	0.75	rs1287276985	C/S	204	probably damaging	0.999
rs368256497	G/W	84	probably damaging	0.926	rs1287276985	C/R	204	probably damaging	0.999
rs374420337	C/Y	86	probably damaging	1	rs1255510121	C/Y	204	probably damaging	0.999
rs368814156	N/D	88	possibly damaging	0.603	rs1229919945	S/P	211	possibly damaging	0.841
rs1421145908	E/K	90	probably damaging	0.981	rs369593555	R/H	212	probably damaging	0.994
rs114262569	R/W	108	probably damaging	0.998	rs369593555	R/L	212	probably damaging	0.987
rs367757959	R/Q	108	probably damaging	0.933	rs1189544809	R/M	216	probably damaging	0.952
rs1284410244	Q/R	113	possibly damaging	0.613	rs749491856	N/H	221	possibly damaging	0.765
rs1456457746	G/R	118	possibly damaging	0.827	rs759826070	E/Q	222	probably damaging	0.991
rs1329473200	Q/R	124	probably damaging	0.914	rs765514291	E/D	222	probably damaging	0.978

rs1412676860	N/H	126	possibly damaging	0.71	rs752907384	R/G	223	probably damaging	0.987
rs1166395511	R/K	131	probably damaging	0.997	rs752907384	R/C	223	probably damaging	0.997
rs76869573	P/T	132	possibly damaging	0.793	rs140461341	R/H	223	probably damaging	0.994
rs760891966	E/V	140	possibly damaging	0.827	rs1272402969	P/A	230	probably damaging	0.942
rs921621484	S/P	143	probably damaging	0.994	rs768366559	P/L	230	probably damaging	0.961
rs1397698749	K/R	149	probably damaging	0.991	rs771561387	R/G	232	probably damaging	0.998
rs1277055983	Q/H	151	probably damaging	0.994	rs771561387	R/W	232	probably damaging	1
rs1475211079	K/T	152	probably damaging	0.996	rs374253522	R/Q	232	probably damaging	0.998
rs775464274	R/Q	153	probably damaging	0.99	rs374253522	R/Q	232	probably damaging	0.998

Table S 3

Results predicted by Cadd

Variant ID	AA coord	Prediction	Score	Variant ID	AA coord	Prediction	Score
rs1208889729	49	likely deleterious	32	rs1287276985	204	likely deleterious	31
rs1267535717	49	likely deleterious	32	rs369593555	212	likely deleterious	31
rs762664023	82	likely deleterious	31	rs866329410	215	likely deleterious	33
rs367757959	108	likely deleterious	31	rs1189544809	216	likely deleterious	32
rs1166395511	131	likely deleterious	34	rs752907384	223	likely deleterious	32
rs760891966	140	likely deleterious	33	rs752907384	223	likely deleterious	32
rs371177206	155	likely deleterious	32	rs748984440	228	likely deleterious	32
rs774265827	155	likely deleterious	31	rs371208770	229	likely deleterious	32
rs774265827	155	likely deleterious	31	rs771561387	232	likely deleterious	34
rs150806902	159	likely deleterious	33	rs771561387	232	likely deleterious	32
rs1003930162	188	likely deleterious	32	rs374253522	232	likely deleterious	33
rs1429874344	190	likely deleterious	32				

Table S 4

Results predicted by Revel

Variant ID	AA	AA coord	Prediction	Score
rs1267535717	R/H	49	likely disease causing	0.611
rs755307045	V/M	59	likely disease causing	0.838
rs748352475	P/A	66	likely disease causing	0.586
rs759253179	V/M	78	likely disease causing	0.881
rs762664023	R/Q	82	likely disease causing	0.84
rs374420337	C/Y	86	likely disease causing	0.708
rs1421145908	E/K	90	likely disease causing	0.566
rs1346131223	T/A	103	likely disease causing	0.74
rs114262569	R/W	108	likely disease causing	0.825
rs756193427	E/A	119	likely disease causing	0.509
rs1329473200	Q/R	124	likely disease causing	0.762
rs1166395511	R/K	131	likely disease causing	0.565
rs76869573	P/T	132	likely disease causing	0.519
rs1003930162	S/L	188	likely disease causing	0.57
rs777632185	R/W	190	likely disease causing	0.587
rs1465046427	K/M	192	likely disease causing	0.517
rs1287276985	C/S	204	likely disease causing	0.702
rs1287276985	C/R	204	likely disease causing	0.574
rs1255510121	C/Y	204	likely disease causing	0.64

Table S 5

Results predicted by MetaLR

Variant ID	AA	AA coord	meta_lr_class	MetaLR
rs755307045	V/M	59	damaging	0.544
rs759253179	V/M	78	damaging	0.549
rs762664023	R/Q	82	damaging	0.502
rs374420337	C/Y	86	damaging	0.549
rs114262569	R/W	108	damaging	0.599
rs367757959	R/Q	108	damaging	0.531
rs1329473200	Q/R	124	damaging	0.545
rs1166395511	R/K	131	damaging	0.549
rs1475211079	K/T	152	damaging	0.74
rs775464274	R/Q	153	damaging	0.515
rs371177206	R/C	155	damaging	0.732
rs774265827	R/H	155	damaging	0.741
rs774265827	R/L	155	damaging	0.741
rs750127977	K/I	157	damaging	0.614
rs267601048	S/F	158	damaging	0.58
rs141138308	R/W	159	damaging	0.753
rs150806902	R/Q	159	damaging	0.706
rs1475279373	G/R	185	damaging	0.55
rs62401172	G/V	185	damaging	0.52
rs1003930162	S/L	188	damaging	0.626
rs777632185	R/W	190	damaging	0.547
rs1429874344	R/Q	190	damaging	0.547
rs1465046427	K/M	192	damaging	0.598

Table S 6

Results predicted by Mutation Assessor

Variant ID	AA	AA coord	Prediction	Score	Variant ID	AA	AA coord	Prediction	Score
rs751447901	F/L	3	low	0.513	rs1357987443	M/L	81	low	0.303
rs757103787	W/R	7	medium	0.702	rs374057152	M/T	81	medium	0.567
rs767279692	H/R	9	medium	0.55	rs762664023	R/Q	82	medium	0.88
rs767279692	H/L	9	medium	0.55	rs368256497	G/R	84	medium	0.792
rs964195968	L/R	12	medium	0.718	rs368256497	G/W	84	medium	0.792
rs1355739548	A/T	13	medium	0.702	rs374420337	C/Y	86	medium	0.909
rs755797583	L/V	16	medium	0.747	rs368814156	N/D	88	medium	0.883
rs755797583	L/F	16	medium	0.747	rs1421145908	E/K	90	medium	0.907
rs748846771	Y/N	17	medium	0.688	rs1459669662	E/G	93	medium	0.853
rs375450405	Y/S	17	medium	0.688	rs1187507161	T/A	97	medium	0.807
rs375450405	Y/C	17	medium	0.688	rs755361036	T/S	97	low	0.411
rs754375185	H/Y	19	medium	0.609	rs755361036	T/I	97	low	0.341
rs1029847964	H/R	20	medium	0.55	rs1334033917	E/K	98	medium	0.697
rs1029847964	H/L	20	medium	0.55	rs758648305	I/T	102	low	0.254
rs1320267753	W/R	23	medium	0.615	rs1346131223	T/A	103	medium	0.916
rs1201834176	P/T	28	medium	0.712	rs114262569	R/W	108	medium	0.866
rs569791806	G/R	34	low	0.292	rs367757959	R/Q	108	medium	0.675
rs761835228	H/Y	38	low	0.383	rs767587788	I/L	109	low	0.352
rs555315943	H/Q	38	low	0.496	rs1284410244	Q/R	113	low	0.513
rs1193943585	E/K	39	low	0.372	rs1456457746	G/R	118	low	0.338
rs1047373716	V/M	41	low	0.383	rs756193427	E/A	119	medium	0.832
rs1357683782	F/I	43	medium	0.755	rs1329473200	Q/R	124	medium	0.729
rs1005693041	V/I	46	medium	0.643	rs1412676860	N/H	126	low	0.345
rs1208889729	R/C	49	medium	0.712	rs1412676860	N/D	126	low	0.21
rs1267535717	R/H	49	medium	0.712	rs1582504137	K/R	127	low	0.323
rs554561071	I/V	55	medium	0.661	rs1166395511	R/K	131	low	0.261
rs375773218	I/M	55	low	0.287	rs76869573	P/T	132	low	0.224
rs1582497910	T/N	57	medium	0.877	rs760891966	E/V	140	low	0.261
rs755307045	V/M	59	high	0.939	rs45533131	R/G	145	low	0.202
rs748352475	P/A	66	medium	0.871	rs1395807781	R/Q	145	low	0.202
rs772184987	P/L	66	medium	0.871	rs1395807781	R/P	145	low	0.202
rs777797068	I/F	69	low	0.454	rs1395807781	R/L	145	low	0.202
rs933893718	E/K	70	medium	0.907	rs1397698749	K/R	149	low	0.245
rs985359727	Y/F	71	low	0.414	rs1277055983	Q/H	151	low	0.261

rs910906787	I/F	72	low	0.451	rs1475211079	K/T	152	low	0.245
rs770795261	I/M	72	medium	0.554	rs775464274	R/Q	153	low	0.261
rs1359856307	K/Q	74	medium	0.76	rs763031344	K/E	154	low	0.261
rs759253179	V/M	78	high	0.944	rs371177206	R/C	155	low	0.245
rs750127977	K/I	157	low	0.261	rs369593555	R/H	212	low	0.479
rs267601048	S/F	158	low	0.224	rs369593555	R/L	212	medium	0.609
rs141138308	R/W	159	low	0.245	rs866329410	A/V	215	low	0.224
rs150806902	R/Q	159	low	0.245	rs1189544809	R/M	216	medium	0.751
rs1003930162	S/L	188	medium	0.75	rs749491856	N/H	221	medium	0.666
rs777632185	R/W	190	medium	0.732	rs759826070	E/Q	222	medium	0.598
rs1429874344	R/Q	190	medium	0.732	rs765514291	E/D	222	medium	0.723
rs1465046427	K/M	192	medium	0.704	rs752907384	R/G	223	medium	0.641
rs1188254133	L/V	194	medium	0.75	rs752907384	R/C	223	medium	0.641
rs376388064	P/L	199	low	0.486	rs140461341	R/H	223	low	0.296
rs776522854	T/M	201	medium	0.775	rs371208770	K/R	229	low	0.394
rs1287276985	C/S	204	medium	0.795	rs1272402969	P/A	230	medium	0.678
rs1287276985	C/R	204	medium	0.795	rs768366559	P/L	230	medium	0.55
rs1255510121	C/Y	204	medium	0.795	rs771561387	R/G	232	medium	0.654
rs769584601	K/R	207	medium	0.56	rs771561387	R/W	232	medium	0.654
rs1229919945	S/P	211	low	0.28	rs374253522	R/Q	232	medium	0.654

Table S 7

Results predicted by PhD-SNP, Panther, Pmut, Mutpred2, SNPs &GO, and SNAP2

Variant ID	AA	AA coord	PhD-SNP	PANTHER	Pdel	Pmut	Score	MutPred 2	Score	SNPs & GO	SNAP 2	Score
rs1208889729	R/C	49	Disease	probably damaging	0.57	Disease	0.90	---	0.43	Disease	effect	49
rs1267535717	R/H	49	Disease	probably damaging	0.57	Disease	0.73	---	0.27	Disease	effect	58
rs755307045	V/M	59	Neutral	probably damaging	0.74	Disease	0.73	Pathogenic	0.61	Disease	effect	42
rs759253179	V/M	78	Neutral	probably damaging	0.74	Disease	0.81	Pathogenic	0.814	Disease	effect	50
rs762664023	R/Q	82	Neutral	probably damaging	0.74	Disease	0.89	Pathogenic	0.705	Disease	effect	79
rs374420337	C/Y	86	Disease	probably damaging	0.74	Disease	0.80	Pathogenic	0.929	Disease	effect	88
rs1421145908	E/K	90	Disease	probably damaging	0.74	Disease	0.73	Pathogenic	0.766	Disease	effect	44
rs114262569	R/W	108	Disease	possibly damaging	0.5	Disease	0.63	Pathogenic	0.531	Disease	effect	79
rs367757959	R/Q	108	Disease	possibly damaging	0.5	Disease	0.68	---	0.334	Disease	effect	56
rs1329473200	Q/R	124	Neutral	probably benign	0.27	Disease	0.85	---	0.289	Disease	effect	8
rs1166395511	R/K	131	Neutral	probably damaging	0.57	Disease	0.73	---	0.223	Disease	effect	44
rs76869573	P/T	132	Disease	probably damaging	0.57	Disease	0.71	---	0.259	Neutral	effect	12
rs371177206	R/C	155	Neutral	probably damaging	0.57	Neutral	0.37	Pathogenic	0.589	Neutral	Neutral	-34
rs774265827	R/H	155	Neutral	probably damaging	0.57	Neutral	0.39	---	0.381	Neutral	Neutral	-40

rs774265827	R/L	155	Neutral	probably damaging	0.57	Neutral	0.43	Pathogenic	0.631	Neutral	Neutral	-14
rs62401172	G/V	185	Neutral	probably benign	0.27	Neutral	0.40	---	0.135	Neutral	Neutral	-40
rs1003930162	S/L	188	Neutral	possibly damaging	0.5	Disease	0.72	---	0.32	Neutral	effect	15
rs777632185	R/W	190	Neutral	possibly damaging	0.5	Disease	0.88	---	0.32	Disease	effect	36
rs1429874344	R/Q	190	Neutral	possibly damaging	0.5	Disease	0.61	---	0.179	Disease	effect	26
rs1465046427	K/M	192	Neutral	probably damaging	0.57	Disease	0.66	---	0.336	Neutral	effect	23
rs1287276985	C/S	204	Disease	probably damaging	0.86	Disease	0.88	Pathogenic	0.829	Disease	effect	70
rs1287276985	C/R	204	Disease	probably damaging	0.86	Disease	0.76	Pathogenic	0.919	Disease	effect	78
rs369593555	R/L	212	Disease	probably damaging	0.57	Disease	0.85	---	0.489	Disease	effect	20
rs1189544809	R/M	216	Neutral	probably damaging	0.57	Disease	0.85	---	0.562	Neutral	effect	32
rs752907384	R/G	223	Disease	probably damaging	0.57	Disease	0.66	Pathogenic	0.754	Disease	effect	61
rs752907384	R/C	223	Disease	probably damaging	0.57	Disease	0.89	Pathogenic	0.714	Disease	effect	30
rs771561387	R/G	232	Neutral	probably damaging	0.57	Disease	0.73	---	0.858	Neutral	effect	51
rs771561387	R/W	232	Neutral	probably damaging	0.57	Disease	0.73	Pathogenic	0.843	Neutral	effect	74
rs374253522	R/Q	232	Neutral	probably damaging	0.57	Disease	0.75	Pathogenic	0.768	Neutral	effect	69

Figure S1: Heat Map indicating pathogenic mutations

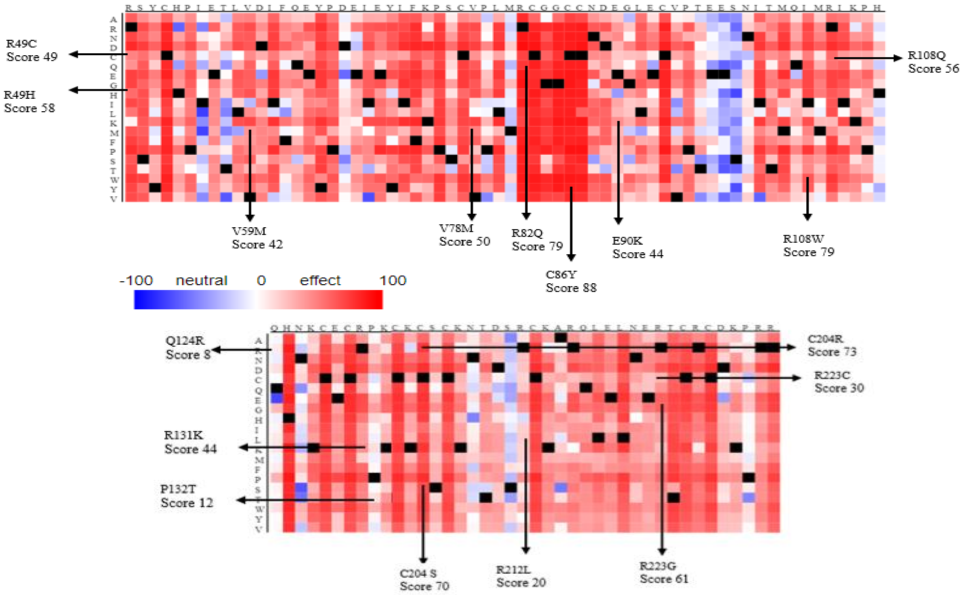

Fig. S 1 Heatmap generated by SNAP 2 showing pathogenic extent of all the mutants

Figure S2 Domains of VEGFA predicted by Interpro Server

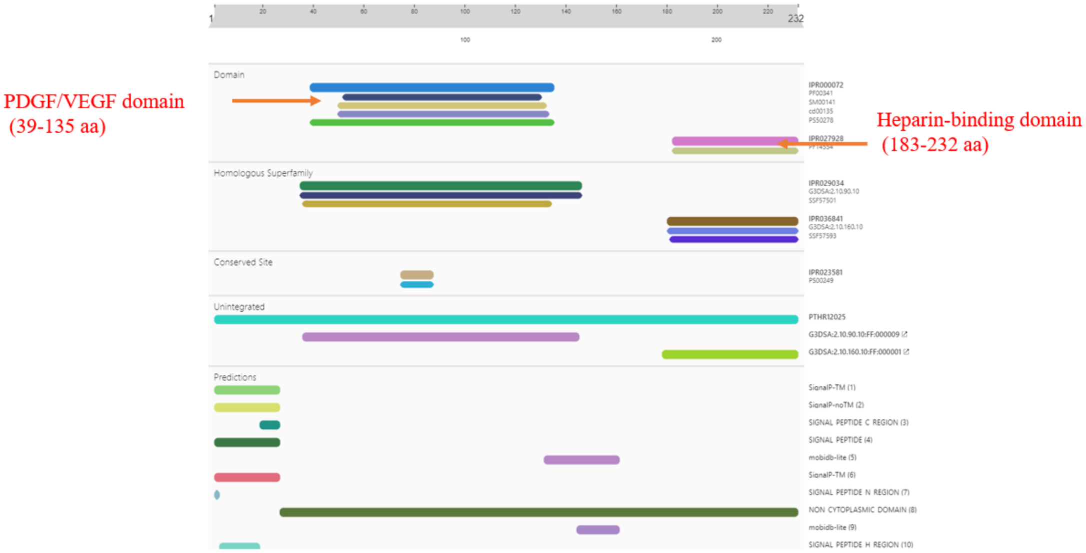

Fig. S 2 Domains of VEGFA identified by Interpro Server. PDGF/VEGF domain is indicated by arrow on the left side. Heparin binding domain is indicated by arrow on right side.

Table S 8

Results predicted by ConSurf

Accession Number	Mutation	Conservation Scale	Effects
**rs1208889729**	R49C	7	exposed residue
**rs1267535717**	R49H	7	exposed residue
**rs755307045**	V59M	9	buried residue/ predicted structural residue
**rs759253179**	V78M	9	buried residue/ predicted structural residue
**rs762664023**	R82Q	9	exposed residue/ predicted functional residue
**rs374420337**	C86Y	9	buried residue/ predicted structural residue
**rs1421145908**	E90K	9	exposed residue/ predicted functional residue
**rs114262569**	R108W	6	exposed residue
**rs367757959**	R108Q	6	exposed residue
**rs1003930162**	S188L	6	exposed residue
**rs777632185**	R190W	7	exposed residue
**rs1429874344**	R190Q	7	exposed residue
**rs1287276985**	C204S	9	buried residue/ predicted structural residue
**rs1287276985**	C204R	9	buried residue/ predicted structural residue
**rs369593555**	R216M	7	exposed residue
**rs752907384**	R223G	7	exposed residue

**rs771561387**<br>**rs771561387**	R232G<br>R232W	8	exposed residue/ predicted functional residue

Table S 9

Predicted change in phenotypic effect of all the mutants of VEGFA determined by HOPE server

**Residue**	**Structure**	**Properties**
**R49C**	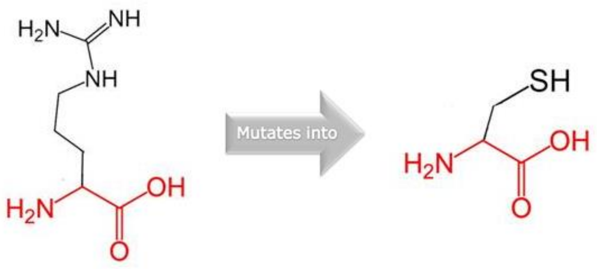	• Charge of wild type residue was positive and when it was mutated with Cystine it became neutral<br>• Size of mutant residue is smaller than wild type.<br>• Mutant residue is hydrophobic
**R49H**	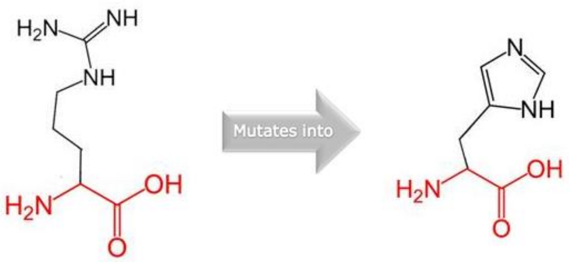	• Size of wild type residue was large but when it was mutated with Histidine its size became smaller.<br>• Wild type residue was positive and the charge of mutant residue is neutral<br>• Substitution of Arginine with Histidine results in small size hence mutated residue might be too small to form multimeric contacts

**V59M**	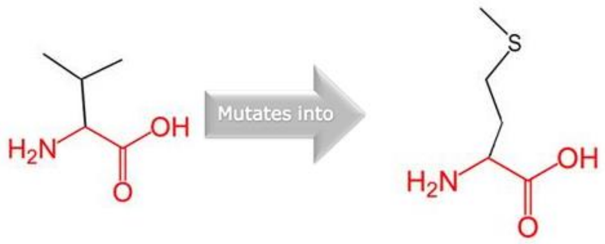	• Size of mutant residue is larger than wild type<br>• Wild-type residue was conserved and buried in protein core. As the size of mutant residue is large it will not easily fit in the core of protein.
**V78M**	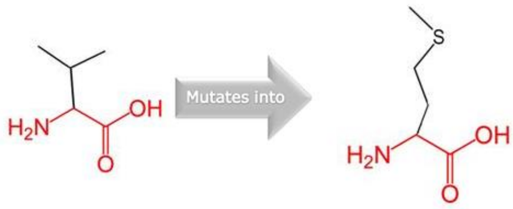	• Size of mutant reside is large as compare to wild type.<br>• The location of mutant residue is very close to a residue that forms cystine bond which can be affected by the mutation if present in its vicinity.<br>• As the size of mutated residue is larger so it can affect multimeric interactions
**R82Q**	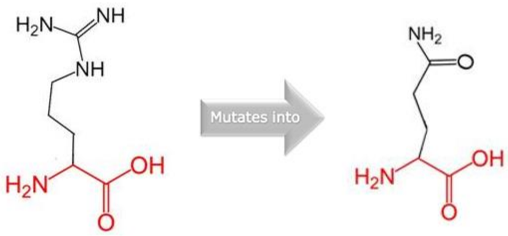	• Size of mutant reside is smaller as compare to wild type.<br>• The charge of wild type residue was positive and when it got mutated with Glutamine its charge became neutral.
**C86Y**	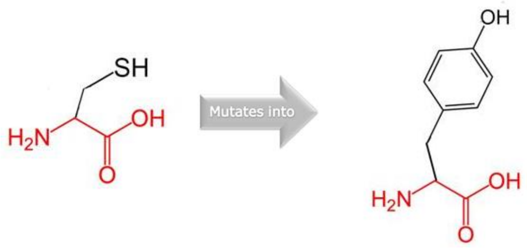	• Size of mutant reside is large as compare to wild type.<br>• The wild-type residue was more hydrophobic than the mutated one hence causing loss of hydrophobic interactions in protein core.
**E90K**	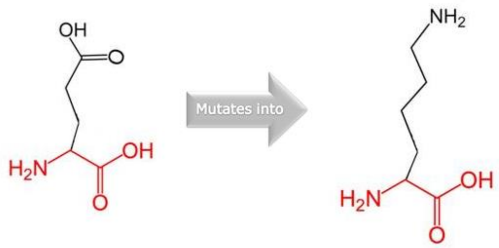	• Size of mutant reside is large as compare to wild type.<br>• Wild type residue was negatively charged and when it got mutated with Lysine it became positively charged hence causing repulsion with other ligands and proteins.

**R108W**	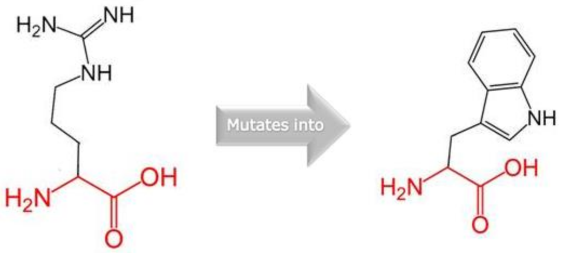	• Size of mutant reside is large as compare to wild type.<br>• The charge of wild type residue was positive, mutation introduced neutral charge to it.<br>• The hydrophobic character of mutant residue got increased as compare to wild type.<br>• The location of wild type residue is on the protein surface and when it got mutated it can affect interactions with other molecules.
**R108Q**	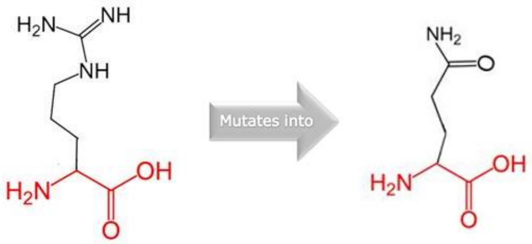	• Size of mutant reside is smaller as compare to wild type.<br>• The charge on wild type residue was positive, when it got mutated with Glutamine it became Neutral

Figure S3: Ramachandran Plots of all nsSNPs

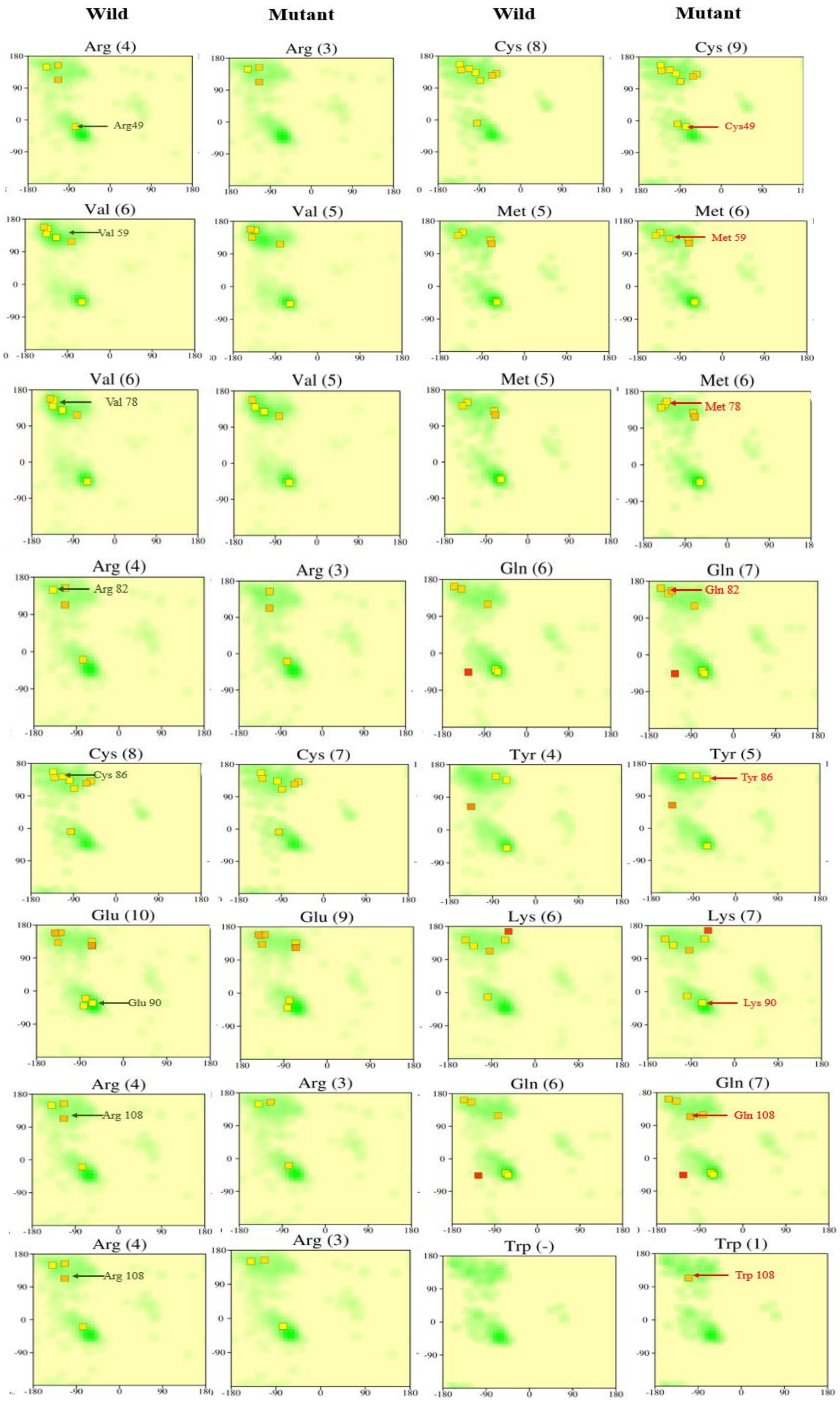

Fig. S 3Constructed Ramachandran Plots for wild and all the mutants

Table S 10

List of conserved interactions that are present in wild showed by all the mutants

Sr#	Wild Type Interactions with VEGFR2	Conserved Interactions							
		R49C	V59M	V78M	R82Q	C86Y	E90K	R108Q	R108W
01	D60 interacting with Q280	✓	-	-	-	-	-	-	✓
02	D67 interacting with K266	✓	-	✓	✓	-	-	✓	-
03	I69 interacting with K266	✓	-	✓	-	-	-	-	-
04	E70 interacting with K266	✓	-	-	✓	-	-	✓	✓
05	E70 interacting with D257	-	-	✓	-	-	-	-	-
06	K110 interacting with D257	✓	-	✓	-	-	-	-	✓
07	H112 interacting with G220	-	-	✓	✓	✓	✓	-	✓
08	H112 interacting with L313	✓	-	✓	✓	✓	✓	✓	✓

Table S 11

List of new interactions formed by all the mutants with IgD2 and IgD3 domains of VEGFR2

New Interactions between VEGFA mutants and VEGFR-2							
**R49C**	**V59M**	**V78M**	**R82Q**	**C86Y**	**E90K**	**R108Q**	**R108W**
Q63 is forming polar contact with T279	E64 is forming polar contact with S153	Q63 is forming polar contact with T279	E70 is forming polar contact with I256	E70 is forming polar contact with H267	I69 is forming polar contact with Q268	Q63 is forming polar contact with K270 and H269	I69 is forming polar contact with I256
D67 is forming polar contact with S265	M59 is forming polar contact with L151	Q63 is forming polar contact with K278	D60 is forming polar contact with K278	E70 is forming polar contact with I256	E70 is forming polar contact with I256	P66 is forming polar contact with Q268	D60 is forming polar contact with T279
E70 is forming polar contact with I256	Q63 is forming polar contact with L151	D60 is forming polar contact with T279	Q115 is forming polar contact with Y137	Q115 is forming polar contact with V218	P66 is forming polar contact with K266	Q115 is forming polar contact with Y137	E70 is forming polar contact with F258
K74 is forming polar contact with N253	T57 is forming polar contact with P149	K74 is forming polar contact with A195	K74 is forming polar contact with A195	K74 is forming polar contact with A195	E70 is forming polar contact with Q268	Q115 is forming polar contact with H133	Q63 is forming polar contact with T279
	E93 is forming polar contact with F185	K74 is forming polar contact with V216	K74 is forming polar contact with V216	K74 is forming polar contact with V216	K74 is forming polar contact with V216	K74 is forming polar contact with V218	K74 is forming polar contact with V216
---------------	Y47 is forming polar contact with K144	---------------	---------------	D67 is forming polar contact with H267	K74 is forming polar contact with A195	E70 is forming polar contact with I256	---------------
	---------------			---------------	D67 is forming polar contact with H267 and Q268	F62 is forming polar contact with K270	

Table S 12

RMSF of Wild and V59M Complexed with VEGFR2

Residue	Position	Wild	V59M	Stability of Mutant compared to wild type
Val	46	0.66	3.16	Fluctuation increased, Stability Decreased
Val/Met	59	0.21	1.60	Fluctuation increased, Stability Decreased
Phe	62	0.20	1.21	Fluctuation increased, Stability Decreased
Tyr	65	0.33	1.24	Fluctuation increased, Stability Decreased
Pro	79	0.18	1.87	Fluctuation increased, Stability Decreased
Met	81	0.26	2.02	Fluctuation increased, Stability Decreased
Asn	88	0.31	2.70	Fluctuation increased, Stability Decreased
Glu	99	0.35	1.53	Fluctuation increased, Stability Decreased
Ser	100	0.29	1.65	Fluctuation increased, Stability Decreased
Ile	117	0.20	1.82	Fluctuation increased, Stability Decreased
Lys	133	0.64	2.31	Fluctuation increased, Stability Decreased

Table S 13

RMSF of Wild and V78M Complexed with VEGFR2

Residue	Position	Wild (nm)	V78M (nm)	Stability of Mutant compared to wild type
Glu	70	0.17	1.34	Fluctuation increased, Stability Decreased
Val/Met	78	0.16	0.85	Fluctuation increased, Stability Decreased
Glu	90	0.46	1.29	Fluctuation increased, Stability Decreased
Ser	100	0.14	0.53	Fluctuation increased, Stability Decreased
Ile	112	0.21	1.58	Fluctuation increased, Stability Decreased
Lys	127	0.39	0.67	Fluctuation increased, Stability Decreased
Lys	133	0.64	1.09	Fluctuation increased, Stability Decreased

Table S 14

RMSF of Wild and R82Q Complexed with VEGFR2

Residue	Position	Wild (nm)	R82Q (nm)	Stability of Mutant compared to wild type
Thr	57	0.22	0.24	Almost similar to wild type
Tyr	65	0.33	0.48	Fluctuation increased, Stability Decreased
Leu	80	0.19	0.22	Almost similar to wild type
Arg/Gln	82	0.23	0.25	Almost similar to wild type
Glu	90	0.46	0.56	Fluctuation increased, Stability Decreased
Ser	100	0.29	0.22	Almost similar to wild type
Gln	113	0.29	0.46	Fluctuation increased, Stability Decreased
Gln	124	0.24	0.21	Almost similar to wild type
Lys	133	0.64	0.56	Fluctuation increased, Stability Decreased

Table S 15

RMSF of Wild and C86Y Complexed with VEGFR2

Residues	Position	Wild (nm)	C86Y (nm)	Stability of Mutant compared to wild type
Glu	54	0.25	0.43	Fluctuation increased, Stability Decreased
Glu	70	0.17	1.61	Fluctuation increased, Stability Decreased
Tyr	71	0.13	1.54	Fluctuation increased, Stability Decreased
Cys/Tyr	86	0.32	0.96	Fluctuation increased, Stability Decreased
Ile	109	0.13	1.54	Fluctuation increased, Stability Decreased
Lys	110	0.14	1.73	Fluctuation increased, Stability Decreased
Pro	111	0.15	1.80	Fluctuation increased, Stability Decreased
His	112	0.21	2.01	Fluctuation increased, Stability Decreased
Gln	113	0.24	1.96	Fluctuation increased, Stability Decreased
Gly	114	0.14	1.73	Fluctuation increased, Stability Decreased
Gln	115	0.23	1.68	Fluctuation increased, Stability Decreased

Table S 16

RMSF of Wild and E90K Complexed with VEGFR2

Residue	Position	Wild (nm)	E90K (nm)	Stability of Mutant compared to wild type
Ser	50	0.38	0.96	Fluctuation increased, Stability Decreased
Phe	62	0.20	2.09	Fluctuation increased, Stability Decreased
Glu	70	0.17	2.43	Fluctuation increased, Stability Decreased
Glu/Lys	90	0.46	1.14	Fluctuation increased, Stability Decreased
Thr	97	0.35	0.88	Fluctuation increased, Stability Decreased
His	112	0.21	2.99	Fluctuation increased, Stability Decreased
Lys	133	0.64	1.30	Fluctuation increased, Stability Decreased

Table S 17

RMSF of Wild and R108Q Complexed with VEGFR2

Residue	Position	Wild (nm)	R108Q (nm)	Stability of Mutant compared to wild type
Glu	39	1.2	0.9369	Fluctuation increased, Stability Decreased
Val	40	1.13	0.94	Almost similar to wild type
Ile	55	0.22	0.39	Almost similar to wild type
Thr	57	0.22	0.37	Almost similar to wild type
Ile	61	0.18	0.26	Almost similar to wild type
Tyr	71	0.13	0.34	Fluctuation increased, Stability Decreased
Arg/Gln	108	0.16	0.35	Fluctuation increased, Stability Decreased
Ile	109	0.13	0.37	Fluctuation increased, Stability Decreased
Pro	132	0.57	0.57	Almost similar to wild type
Lys	133	0.64	0.73	Almost similar to wild type

Table S 18

RMSF of Wild and R108W Complexed with VEGFR2

Residue	Position	Wild (nm)	R108W (nm)	Stability of Mutant compared to wild type
Cys	52	0.23	0.69	Fluctuation increased, Stability Decreased
Phe	62	0.20	1.01	Fluctuation increased, Stability Decreased
Tyr	71	0.13	1.09	Fluctuation increased, Stability Decreased
Ile	72	0.14	1.14	Fluctuation increased, Stability Decreased
Arg/Trp	108	0.16	1.00	Fluctuation increased, Stability Decreased
Ile	109	0.13	1.16	Fluctuation increased, Stability Decreased
His	112	0.21	1.47	Fluctuation increased, Stability Decreased
